# Prediction of Oswestry Disability Index and Numeric Rating Scale scores after lumbar spine surgery: machine learning model development and fairness assessment

**DOI:** 10.1136/bmjopen-2025-108947

**Published:** 2026-05-13

**Authors:** Henrik Lykke Joakimsen, Jørgen Aarmo Lund, Joel Burman, Ashenafi Zebene Woldaregay, Bjørnar Berg, Tore K Solberg, Tor Ingebrigtsen, Karl Øyvind Mikalsen

**Affiliations:** 1 Department of Clinical Medicine, UiT The Arctic University of Norway, Tromsø, Troms, Norway; 2 The Norwegian Registry for Spine Surgery and the Department of Neurosurgery, University Hospital of North Norway, Tromsø, Troms, Norway; 3 Machine Learning Group, Faculty of Science and Technology, UiT The Arctic University of Norway, Tromsø, Troms, Norway; 4 The Norwegian Centre for Clinical Artificial Intelligence, University Hospital of North Norway, Tromsø, Troms, Norway; 5 DIPS AS, Bodø, Nordland, Norway; 6 Centre for Intelligent Musculoskeletal Health, Oslo Metropolitan University, Oslo, Oslo, Norway

**Keywords:** Artificial Intelligence, Clinical Decision-Making, Spine

## Abstract

**Abstract:**

**Background:**

One-third of patients operated for degenerative conditions in the lumbar spine do not report substantial improvement after 12 months. Most previous outcome prediction models are classifiers. This constrains nuances in prediction and use for decision support.

**Objectives:**

To develop and test models for the prediction of continuous outcome scores and retrieval of similar patients’ outcomes, and to evaluate the models’ fairness.

**Setting:**

Norwegian public and private specialist healthcare.

**Participants and data source:**

All cases recorded with an elective operation for lumbar disc herniation (LDH, n=18 377) or lumbar spinal stenosis (LSS, n=24 540) in the Norwegian Registry for Spine Surgery from 1 January 2007 to 23 May 2023.

**Outcome measures:**

All outcomes were patient-reported 12 months after the operation. The primary outcome was the Oswestry disability index (ODI), modelled on a scale ranging from 0 to 100. Numeric Rating Scale scores (range 0–10) for back and leg pain were secondary outcomes.

**Model building and performance:**

We selected 22 predictors recorded preoperatively by patients and clinicians based on Shapley Additive Explanations values. Data were split into 80%/20% training/test samples for LDH and LSS. Six machine learning methods for regression, that is, with a continuous outcome (extreme gradient boosting (XGBoost), Gaussian process regression, gradient boosting regression, artificial neural networks and linear regression), were trained for both conditions using fivefold cross-validation. We report the magnitude and distribution of errors as mean absolute error (MAE) with 95% CIs, and explanatory power as the coefficient of determination (R^2^). Fairness and calibration were assessed with violin and calibration plots of error. We developed a patient-similarity function that uses a K-nearest neighbour model to retrieve the individual outcomes of the 50 most similar patients and evaluated it by calculating L1 distances (Manhattan distances) across subgroups.

**Results:**

XGBoost regression performed best for both conditions. The models showed good calibration and predicted ODI with MAE 11.32 (95% CI 11.00 to 11.63) and R^2^ 0.27 (95% CI 0.24 to 0.29) for LDH and MAE 12.05 (95% CI 11.76 to 12.32) and R^2^ 0.31 (95% CI 0.28 to 0.34) for LSS. The MAEs for back and leg pain were 2.09 (95% CI 2.04 to 2.15) and 1.95 (95% CI 1.90 to 2.00) for LDH and 2.33 (95% CI 2.28 to 2.38) and 2.13 (95% CI 2.08 to 2.16) for LSS. All models were fair with differences in error between subgroups for sex, age, education level and native language. In the patient-similarity function, distances at baseline were evenly distributed across subgroups.

**Conclusions:**

Our machine learning models predicted continuous outcomes with MAEs close to the SEs of measurements. The models were fair across sociodemographic subgroups. We succeeded in developing a patient-similarity function which supplements the predictions.

**STRENGTHS AND LIMITATIONS OF THIS STUDY**

- The models predict outcomes on continuous scales, avoiding misclassification problems related to predefined outcome categories (eg, success vs non-success).
- The study conducted a fairness analysis showing performance with respect to differences in *age, education level*, *sex* and *native language*.
- The study also developed a patient-similarity function which retrieves and reports individual outcomes of similar cases.
- The models only predict outcomes of surgical treatment and do not compare them with outcomes of non-surgical treatment.

## Introduction

 Lumbar disc herniation (LDH) and lumbar spinal stenosis (LSS) are major causes of health loss and disability worldwide.^1^,^3^ Surgical treatment is effective for selected cases. According to the Norwegian Registry for Spine Surgery (NORspine) and other international reports, only two-thirds of those who receive surgery experience substantial improvement of pain-related disability.^4^,^6^

Well-informed shared decision-making based on accurate individual outcome prediction can improve surgical outcomes by facilitating the selection of patients who will benefit from operative treatment.^7 8^ Numerous classifier models for the prediction of dichotomous outcomes have been developed for spine surgery.^79^,^12^ Several are artificial intelligence (AI)-enabled.^13^,^16^ Unfortunately, most published models have been limited by small sample sizes. In 2021, we succeeded in developing a well-performing classifier model trained on a large dataset from NORspine.^17^ However, a problem with classifiers is their dependence on hard cut-offs, which constrains precision by diminishing the nuances of a prediction. Classifiers do not indicate where on the spectrum from ‘success’ to ‘non-success’ a prediction falls. This is problematic, since different patients may perceive the same degree of improvement very differently. Fixed outcome categories are also inherently prone to misclassification.

Further, in consultations on whether to operate or not, understanding and communicating the probability of improvement and risks can be challenging. Providing accessible, empirical information about the surgical outcomes of similar patients, such as the percentage achieving substantial improvement or worsening, could be a valuable supplement in informing shared decision-making.^18^

An important concern regarding the use of machine learning is that models can perpetuate existing societal biases, sometimes in an unpredictable manner. Subgroups of patients can be subject to unfair or discriminatory predictions. This can be due to imbalanced training data, where minorities, by definition, are less represented. Consequently, assessment of fairness and equality in model performance across subgroups is strongly recommended.^19^ To our knowledge, no previous studies on outcome prediction after spine surgery have presented such evaluations.^20^

### Objectives

The objectives of this study were to develop models for the prediction of continuous outcome scale scores after lumbar spine surgery, to evaluate their fairness and to develop empirical models for the retrieval of similar patients’ outcomes.

## Methods

This study aligns with the recommendations of transparent reporting of a multivariable prediction model for individual prognosis or diagnosis statement for reporting clinical prediction models that use machine learning (TRIPOD+AI).^19^

### Data source

We used data from NORspine, which comprises a cohort of 69 718 cases registered across all 41 public and private hospitals in Norway providing spine surgery. It had a capture rate of 82%, a 3-month follow-up rate of 80% and a 12-month follow-up rate of 72% in 2023.^21^ A drop-out analysis at baseline showed a lower (34%) capture rate for patients undergoing acute rather than scheduled operations and a 3.0-year lower mean age for non-captured than captured cases but no sex difference.^22^ A published drop-out analysis at 12 month follow-up showed that non-responders were 4.7 years younger than responders and that a higher proportion (30% vs 21%) were smokers, but there were no differences in outcomes.^23^ Patients lost to follow-up are thus considered to be missing at random.

The registry includes patients operated on for degenerative disorders in the lumbosacral spine, except patients unable to consent due to age (<16 years), severe psychiatric disorders, substantial drug use, cognitive impairment or language barriers. Patients operated on for tumours, trauma, primary infection or non-degenerative scoliosis are also excluded. The most common surgical indications in 2023 were LSS (47.9%) and LDH (36.6%).^21^

The registry contains a large set of data on patients’ characteristics, process measures and patient-reported outcome measures (PROMs) collected at baseline (before surgery) and at 3 and 12 months after surgery. Self-reporting bias is limited by NORspine’s use of validated and responsive PROMs recommended by the International Consortium for Health Outcomes Measurement.^24^ The questionnaires record the same PROMs at different timepoints to minimise recall bias. Reoperation within 90 days is recorded as treatment of a complication, while new spine surgery after 90 days is considered a new case and initiates new 3-month and 12-month follow-ups.^25^

### Participants

**Figure 1:**
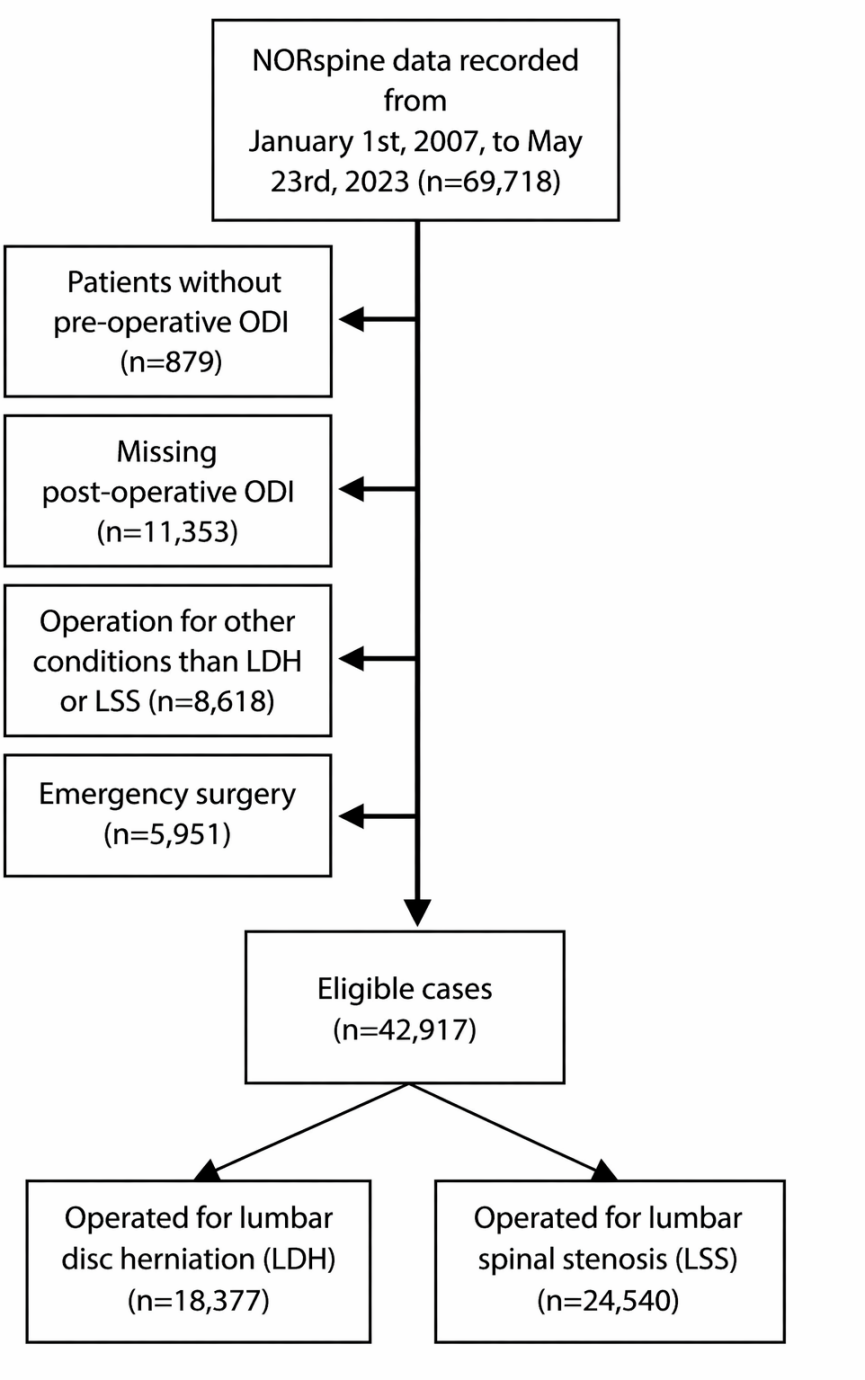
Flowchart of participants in modelling of ODI 12 months after surgery. LDH, lumbar disc herniation; LSS, lumbar spinal stenosis; NORspine, the Norwegian Registry for Spine Surgery; ODI, Oswestry Disability Index.

Figure 1 shows that we retrieved baseline data for all cases recorded in NORspine from 1 January 2007 to 23 May 2023 and corresponding outcome data reported until 23 November 2024. We included cases with MRI-confirmed LDH or LSS who underwent elective decompression surgery with or without fusion. We excluded patients undergoing operations recorded as acute by the surgeon. This includes treatment of new or rapidly progressing neurologic deficits. Patients with missing outcome data were excluded listwise, that is, missing preoperative (n=879) or postoperative (at both 3-month and 12-month follow-up) (n=11 353) Oswestry Disability Index (ODI) scores, back pain (preoperative n=3669, postoperative n=10 954) or leg pain (preoperative n=4235, postoperative n=10 975) scores.

### Data preparation

Before preprocessing, the data were divided into one set for LDH and one for LSS. All the following steps were done identically for both datasets: cases with height outside a range of 130–220 cm (n=20), Body Mass Index outside 10–50 kg/m^2^ (n=47), weight outside 25–250 kg (n=2) and age below 16 years or above 100 years (n=43) were excluded.

Ordinal features were ordered so that logically related options are adjacent; for instance, civil status was coded with value 1 for married, 2 for cohabiting and 3 for living alone. This ensured that similar cases were grouped together in the coordinate space. To be able to compare the different models fairly, we formatted the data into one common format that worked for all methods. Therefore, ordinal features with more than two categories were ordered and treated as continuous. Nominal data were treated as dummy variables (dichotomised for each alternative), which were then treated as continuous.

### Outcome

The primary outcome was ODI 12 months after the operation.^26^ The ODI ranges from 0 to 100 (no disability to maximum disability) and is a summary of 10 items. Each item is graded on a 0–5-point Likert scale assessing pain, self-care, lifting, walking, sitting, standing, sleeping, sexual life, social life and travelling. We also performed a sensitivity analysis using classification with strict criteria for the definition of success (decrease in postoperative ODI≥22 for LDH and ≥14 points for LSS). The cut-offs were validated according to anchor-based predictive modelling^27^ and differ slightly from the cut-offs used in our earlier work.^25 28^

Secondary outcomes were back and leg pain scored on 11-point Numeric Rating Scales (NRS) ranging from 0 to 10 (no pain to worst imaginable pain).^29^ These outcomes were modelled on continuous scales only.

### Predictors

All available presurgical features in NORspine (listed in online supplemental table S1 in the supplementary file) were assessed as potential predictors. Initially, assessment was conducted by training models using all presurgical features. We then measured each feature’s contribution to the final predictive model by its Shapley Additive Explanations (SHAP) values, with the method proposed by Lundberg and Lee.^30^ From this, a subset of candidate features was selected and reviewed with experienced spine surgeons (TKS and TI) and a lawyer (MKH) regarding clinical relevance and legality in the potential subsequent decision support product development.

### Sample size

For sample size calculations, we targeted adequate shrinkage (≥0.9), low optimism (≤0.05) and adequate precision of model estimates with an adjusted coefficient of determination (R^2^) ≥0.20, with an SD of 16.9 (LSS) and 17.6 (LDH) and a mean outcome (ODI at 12 months) of 24.0 (LSS) and 22.0 (LDH), assuming a multiplicative margin of error of 10% and using up to 50 degrees of freedom. This corresponded to a requirement of a minimum of 1924 cases for training for both LDH and LSS. The expected R^2^ was decided empirically, influenced by the results of earlier classifiers and a linear regression model published in 2020.^31^ Intercepts and SD were based on earlier reported mean post-operative ODI.^32^ Sample size calculations were done using the ‘pmsampsize’^33^ package in R Statistical Software (V.9.1.2; R Core Team, 2024).

### Missing data

Previous studies showed a very low proportion of missing data in NORspine, ranging from 0.0% to 6.9% at baseline and from 0.3% to 3.6% at 12-month follow-up for the different variables and found that missingness was compatible with a missing-at-random assumption.^23 25^ There are some instructions and definitions in the registry that are useful to be aware of when assessing missing data. The degree of paresis is assumed to be normal if missing. If information about whether a patient has applied for disability benefits is missing, it is assumed they have not applied. Otherwise, missing data were imputed using a K-nearest neighbour (KNN) imputer.^34^ After initial experiments, values of K in the range 3–200 were found satisfactory and further tested to find an optimal threshold. Optimal K was chosen by balancing performance and complexity based on repeated experiments and expert assessment. We weighted towards lower K, that is, where the curve of performance flattened. Finally, we compared models trained on imputed data to models trained without imputation, as well as to models trained on data imputed with the mean value of each feature. For cases with available 3-month but missing 12-month scores (ODI n=5879, back pain n=5642 and leg pain n=5698), imputation was done using last observation carried forward. The impact of this assumption was evaluated in sensitivity analyses by comparing models trained on complete cases only with models trained using last-observation-carried-forward imputation. Imputed cases were not used in the test set.

### Analytical methods

#### Prediction of continuous outcomes

To validate model structures, each dataset was divided into an 80%/20% training/test split. All variables in both datasets were then standardised using the formula:

$z=\frac{x-u}{s}$ where *z* is the standardised score, *x* is the raw score, *u* is the mean and *s* is the SD of the training samples only.

There was no difference in preprocessing steps across different sociodemographic groups, that is, age, education level, sex or native language. Fivefold cross-validation was used on the training data to train six different statistical and AI-enabled methods: linear regression, gradient boosting regression, extreme gradient boosting (XGBoost), Gaussian process regression and artificial neural networks.

Using the test data, the models were evaluated for the prediction of ODI and NRS scores for back and leg pain 12 months after the operation. As regression models can give predictions outside the logical ranges of ODI 0–100 and NRS 0–10, out-of-range predictions were set to a minimum of 0 or a maximum of 100 for ODI and 10 for NRS.

#### Hyperparameter tuning and evaluation

We only report the exact details of training, tuning and performance of the top-performing models according to mean absolute error (MAE). They were XGBoost with KNN imputation of missing data for both LDH and LSS cases for ODI, back pain and leg pain. Hyperparameter tuning for the XGBoost models was done with a grid search over the following parameters:

- Learning rate: from 0.0001 to 1.0.
- Max depth: 1–4.
- Number of estimators: 50, 100, 200, 300 and 500.

Models were evaluated using a squared error loss function. For the remaining hyperparameters, we used the default values from the XGBoost package.^35^ For performance evaluation, we report MAE and R^2^. Calibration was assessed visually. We used the following definitions from^36^:


$$MAE=\frac{1}{n}\sum_{i=1}^{n}1/2Y_{i}-{\hat{y}}_{i}1/2$$

where *n* is the number of observations in the dataset, *y*_*i*_ is the true value and *ŷ*_*i*_ is the predicted value.

R^2^ is defined as $R^{2}=1-\frac{{SS}_{res}}{{SS}_{tot}}$

where SS_res_ is the sum of squared residuals and SS_tot_ is the total sum of squares.

#### Sensitivity analyses

We tested the regression models in a classification setting because models predicting continuous outcomes have few comparators in the literature. This was done by reinterpreting regression output as probabilities under the assumption that a larger estimated difference *r* between preoperative and postoperative ODI can be interpreted as a greater probability of success, $\hat{p}$:


$$\hat{p}(r)=\frac{r-\min(r)}{\max(r)-\min(r)}=\frac{r-(-100)}{200}$$

This also made it possible to calculate the area under the receiver operating curve (AUC) for the regression models when used for classification. We calculated the AUC as the proportion of cases where a patient with an outcome classified as successful would have a higher estimated probability of a successful outcome than a patient without, using the formula:


$$AUC(f)=\frac{\sum_{s\in S}\sum_{n\in N}1[\hat{p}(s)§gt;\hat{p}(n)]}{|S|\cdot |N|}$$

where *S* and *N* are the patients who achieved successful and non-successful outcomes, respectively.^37^

Van den Goorbergh *et al*^38^ and Carriero *et al*^39^ found that class imbalance correction methods can lead to overestimation of risk and worse calibration. Considering this, the class imbalance was not considered severe enough to warrant using imbalance correction methods.

#### Patient similarity

We also developed an empirical comparison function that provides outcomes of similar cases. The function reports the individual ODI and NRS back and leg pain scores of similar cases 12 months after the operation.

This works by providing the new case to a KNN model, which ranks the similarity of all previous cases at baseline by exploring L1 distance,^40^ also known as Manhattan distance, in feature space. To put cases into a common, continuous coordinate space, we applied the feature preprocessing methods for the regression model for these models as well. The model uses the same features as the regression model but scales them by their SHAP importance, requiring greater similarity for the features which are more important to the model. The SHAP importance scores were derived by calculating the mean SHAP values for each input feature from the regression model applied to the test set, normalising these means and multiplying them by the number of features in the model. To ensure consistency in crucial features, some constraints were applied: age and ODI were restricted to ±5 years and score ±5 points, and sex was matched exactly. The threshold for K was decided empirically.

### Fairness

Fairness of the regression models was assessed by comparing performance (mean error and distribution of error) across demographic subgroups for sex, age, education and native language.

### Model output

The main outcome (ODI) was modelled with regression on a continuous scale. For sensitivity analyses, we report how models performed for the classification of success/non-success. Secondary outcomes (NRS back and leg pain) were modelled on continuous scales only.

### Training versus evaluation

There were no differences between the training and test data in terms of healthcare setting, eligibility criteria, outcomes or features.

### Patient and public involvement

The study’s rationale and design were presented to and discussed with patients’ representatives from the Norwegian Spine Patients’ Association (Ryggforeningen) and spine surgeons’ representatives during NORspine’s advisory board and annual stakeholder meetings.

## Open science

### Protocol

No prospective protocol was published for this study.

### Registration

This study was not registered.

### Code sharing

Analytical codes are available on request.

## Results

### Participants

Figure 1 shows that we screened 69 718 cases. For ODI prediction, we included 18 377 and 24 540 cases operated on for LDH and LSS, respectively. Table 1 shows baseline characteristics of included cases with available ODI at follow-up and of cases excluded due to missing ODI. An extended table for baseline characteristics is found in the online supplemental table S2. For NRS back pain prediction, we included 17 913 LDH cases and 23 089 LSS cases, while for NRS leg pain prediction, we included 17 897 LDH cases and 22 763 LSS cases. Onlinesupplemental tables S3  S4 show baseline characteristics for cases with and without available NRS leg and back pain scores at follow-up.

**Table 1:** Baseline characteristics of cases included in the ODI model development

Variables at baseline	LDH			LSS		
	Included (n=18 377)		Excluded cases due to no follow-up ODI (n=4753)	Included (n=24 540)		Excluded cases due to no follow-up ODI (n=3579)
	Cases with available 12-month ODI (n=15 398)	Cases with available 3-month, but not 12-month ODI (n=2979)		Cases with available 12-month ODI (n=21 640)	Cases with available 3-month, but not 12-month ODI (n=2900)	
Sex, n						
*Missing* (%)	*0* (*0*)	*0* (*0*)	*0* (*0*)	*0* (*0*)	*0* (*0*)	*0* (*0*)
Female (%)	6629 (43.1)	1194 (40.1)	1669 (35.1)	11 059 (51.1)	1504 (51.9)	1759 (49.1)
Male (%)	8769 (56.9)	1785 (59.9)	3084 (64.9)	10 581 (48.9)	1396 (48.1)	1820 (50.9)
Age, mean (SD)	49.09 (14.40)	44.44 (13.54)	41.52 (12.70)	66.28 (11.29)	63.86 (13.14)	61.01 (13.67)
*Missing, n* (%)	*41* (*0.3*)	*15* (*0.5*)	*22* (*0.5*)	*33* (*0.2*)	*2* (*0.1*)	*11* (*0.3*)
Smoking status, n						
*Missing* (%)	*131* (*0.9*)	*24* (*0.8*)	*51* (*1.1*)	*205* (*0.9*)	*31* (*1.1*)	*44* (*1.2*)
Yes (%)	3283 (21.3)	846 (28.4)	1426 (30.0)	3690 (17.1)	646 (22.3)	982 (27.4)
No	11 984 (77.8)	2109 (70.8)	3276 (68.9)	17 745 (82.0)	2223 (76.7)	2553 (71.3)
BMI, mean (SD)	26.87 (4.33)	27.09 (4.51)	27.21 (4.53)	27.68 (4.41)	27.69 (4.61)	27.99 (4.71)
*Missing, n* (%)	*864* (*5.6*)	*175* (*5.9*)	*251* (*5.3*)	*891* (*4.1*)	*124* (*4.3*)	*166* (*4.6*)
NRS back pain, mean (SD)	6.12 (2.36)	6.23 (2.33)	6.19 (2.29)	6.52 (2.17)	6.69 (2.15)	6.64 (2.18)
*Missing, n* (%)	*424* (*2.8*)	*69* (*2.3*)	*166* (*3.5*)	*1356* (*6.3*)	*231* (*8.0*)	*252* (*7.0*)
NRS leg pain, mean (SD)	6.73 (2.15)	6.75 (2.14)	6.67 (2.13)	6.56 (2.22)	6.76 (2.19)	6.61 (2.25)
*Missing, n* (%)	*410* (*2.7*)	*79* (*2.7*)	*167* (*3.5*)	*1573* (*7.3*)	*274* (*9.4*)	*289* (*8.1*)
ODI, mean (SD)	42.78 (16.71)	42.95 (17.11)	42.57 (16.90)	38.61 (14.91)	40.71 (15.15)	41.51 (15.68)
*Missing, n* (%)	*0* (*0*)	*0* (*0*)	*0* (*0*)	*0* (*0*)	*0* (*0*)	*0* (*0*)
Has other relevant diseases, n						
*Missing* (%)	*1120* (*7.3*)	*249* (*8.4*)	*333* (*7.0*)	*1120* (*5.2*)	*132* (*4.6*)	*199* (*5.6*)
Yes (%)	5141 (33.4)	900 (30.2)	1342 (28.2)	14 119 (65.2)	1875 (64.7)	2173 (60.7)
No (%)	9137 (59.3)	1830 (61.4)	3078 (64.8)	6401 (29.6)	893 (30.8)	1207 (33.7)

BMI, Body Mass Index; LDH, lumbar disc herniation; LSS, lumbar spinal stenosis; NRS, Numeric Rating Scale; ODI, Oswestry Disability Index.

Descriptions of all included predictors for the ODI models (after imputation and preprocessing) are listed in table 2.

**Table 2:** Predictors included in the ODI models

Predictor	Baseline value for LDH cases			Baseline value for LSS cases			Unit of measure (explanation)
	Missing n (%)	Mean (SE)	SHAP score	Missing n (%)	Mean (SE)	SHAP score	
Age	56 (0.3)	48 (14)	0.9	34 (0.1)	66 (12)	1.0	Age in years
Smoking status	154 (0.8)	0.5 (0.8)	0.8	228 (1.0)	0.4 (0.8)	0.4	0, 1 (no, yes)
BMI	1028 (5.7)	27 (4.4)	0.6	989 (4.2)	28 (4.4)	0.4	kg/m^2^
Symptom duration (leg pain)	592 (3.3)	3.5 (1.0)	2.2	1074 (4.5)	4.2 (1.0)	1.7	1–5*
Symptom duration (back/hip pain)	735 (4.1)	3.2 (0.9)	1.0	1471 (6.1)	3.9 (1.1)	0.9	1–5*
Use of painkillers	69 (0.4)	0.8 (0.4)	0.0	150 (0.7)	0.8 (0.4)	0.3	0, 1 (no, yes)
NRS back/hip pain	482 (2.7)	6.1 (2.4)	1.1	1510 (6.4)	6.5 (2.2)	0.5	Scale (1–10)
NRS leg pain	472 (2.7)	6.7 (2.1)	0.7	1755 (7.5)	6.6 (2.2)	0.4	Scale (1–10)
ODI	0 (0.0)	43 (17)	2.7	0 (0.0)	39 (15)	5.1	Scale (1–100)
EQ5D index	585 (3.3)	0.4 (0.3)	0.4	1296 (5.4)	0.4 (0.3)	0.4	Index (−0.59 to 1, calculated using UK values)
EQ5D anxiety score	249 (1.4)	1.9 (1.1)	1.3	600 (2.5)	1.7 (1.0)	1.0	1–5 (low→high)
EQ VAS health score	837 (4.8)	47 (21)	0.3	1820 (7.5)	50 (20)	0.4	Scale (1–100, worst→best)
Civil status	149 (0.9)	0.8 (0.4)	0.1	228 (1.0)	0.7 (0.4)	0.2	1–3 (married, cohabiting, living alone)
Work status	587 (2.6)	1.5 (1.1)	1.2	1208 (4.1)	1.3 (1.0)	0.5	1–9†
Education level	213 (1.2)	1.4 (1.0)	1.0	769 (3.3)	1.3 (1.0)	0.8	1–5‡
Applied for disability benefits	1182 (6.7)	0.3 (0.9)	0.7	5380 (23)	0.6 (1.2)	0.6	1–4 (yes, no, planning to, got it)
Previously operated	100 (0.6)	0.2 (0.4)	0.8	168 (0.7)	0.2 (0.4)	1.1	1–4 (same level, another level, same and another level, no)
Number of previous operations	462 (2.6)	0.3 (0.6)	1.0	702 (2.9)	0.3 (0.7)	1.2	Number (0–999)
Other relevant diseases	1366 (7.5)	0.4 (0.5)	0.1	1251 (5.1)	0.7 (0.5)	0.0	0, 1 (no, yes)
Degree of paresis	15 292 (85)	3.9 (0.8)	0.0	22 042 (93)	4.0 (1.0)	0.0	0–5 (paralysed, level 1, level 5 equal to normal strength)
ASA score	227 (1.2)	1.6 (0.6)	0.5	336 (1.4)	2.1 (0.6)	0.5	1–5 (level 1, level 5, healthy→sick)
Sex	0 (0.0)	0.6 (0.5)	0.5	0 (0.0)	0.5 (0.5)	0.0	0, 1 (female, male)

* 1, no symptoms; 2, 0–3 months; 3, 3–12 months; 4, 12–24 months and; 5, more than 24 months.

† 1, full-time job; 2, part-time job; 3, student; 4, retired; 5, unemployed; 6, on sick leave; 7, partially on sick leave; 8, work settlement allowance and 9, disability pension.

‡ 1, elementary school, 7–10 years; 2, high school vocational path; 3, high school general subject; 4, university<4 years and 5, university>5 years.

ASA, American Society of Anaesthesiologists; BMI, Body Mass Index; EQ, EuroQol; EQ5D, EuroQol five-dimensions; LDH, lumbar disc herniation; LSS, lumbar spinal stenosis; NRS, Numeric Rating Scale; ODI, Oswestry Disability Index; SHAP, Shapley Additive Explanations; VAS, Visual Analogue Scale.

### Model development

#### Prediction of continuous outcomes

After SHAP analyses and expert discussions, a subset of the 22 predictors with the highest SHAP values was chosen (listed in table 2). Adding more predictors gave no significant increase in performance for any of the models. For ODI, the final XGBoost regression models used the following hyperparameters:

- LDH: learning rate=0.1, max depth=4, number of estimators=100.
- LSS: learning rate=0.1, max depth=3, number of estimators=200.

Hyperparameters for the NRS models are listed in online supplemental table S5 in the supplementary file.

#### Patient similarity

The number K=50 (similar cases) was chosen discretely to strike a balance between precision in finding similar cases and sufficient data for comparison with such cases, after discussion between computer scientists (JAL, JB, KØM and AZW) and surgeons (TKS and TI).

### Model specification

The regression models were packaged in the XGBoost model format, with preprocessing implemented in Python. The patient-similarity system was built on top of scikit-learn’s implementation of K-dimensional trees, with a Python wrapper to implement bracketing by age, sex and presurgical ODI. The algorithms were developed with Python V.3.11.9, XGBoost V.1.7.3 and scikit-learn V.1.4.1.

The inference and training code, as well as the regression algorithms, are available on request. The dataset, including the subset used for patient similarity, can only be shared with approval from NORspine. The project’s data processing agreement only permits processing of the data within the University Hospital of North Norway’s IT systems.

### Model performance

#### Prediction of continuous outcomes

**Figure 2:**
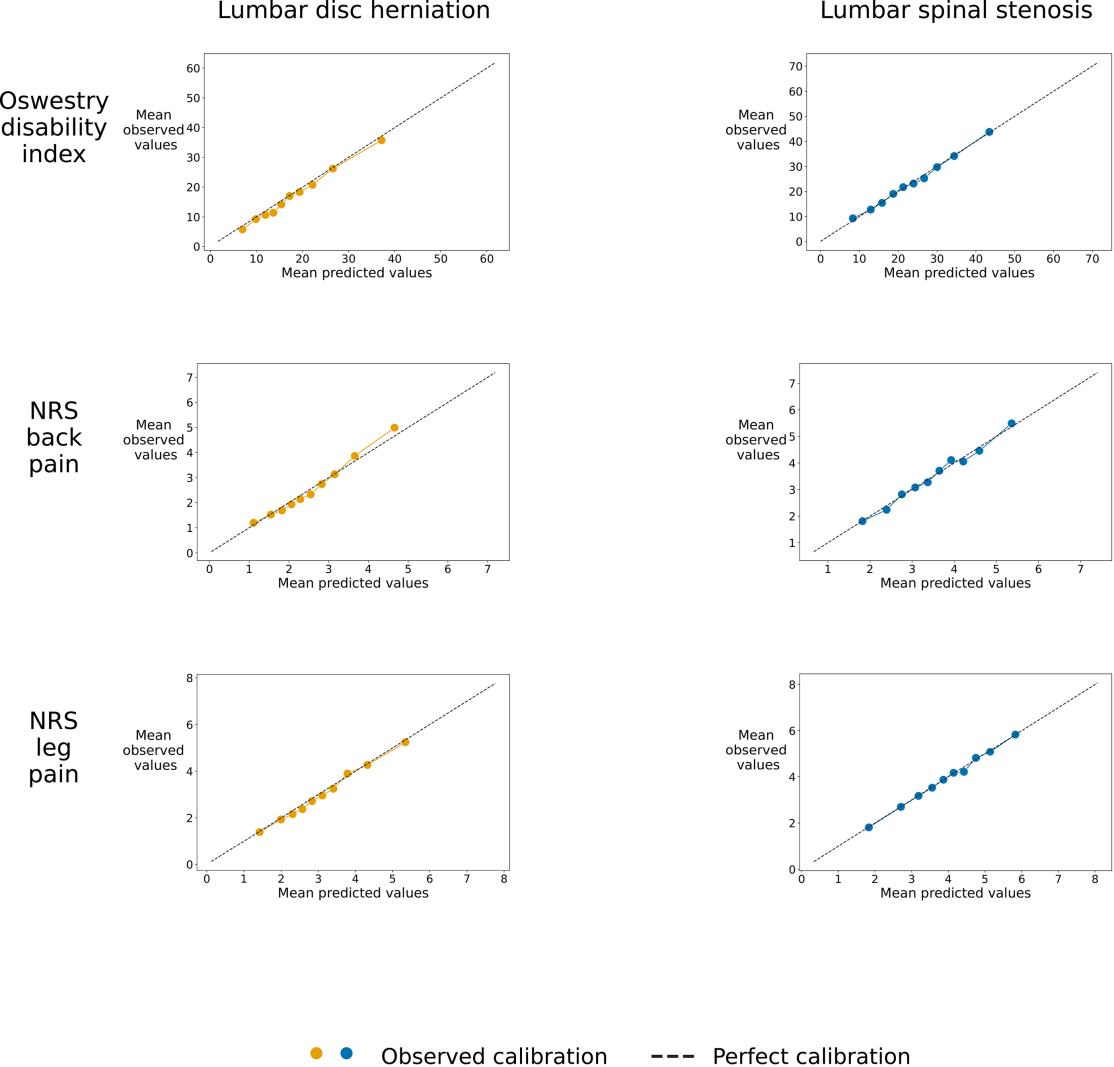
Calibration plots. Calibration plots showing observed versus predicted values for all models. The coloured dots and line represent our models, while the grey dashed line shows what a perfect calibration would be. NRS, Numeric Rating Scale.

Table 3 shows that the models achieved MAE 11.32 (95% CI 11.00 to 11.63) with R^2^ 0.27 (95% CI 0.24 to 0.29) for LDH cases and MAE 12.05 (95% CI 11.76 to 12.32) with R^2^ 0.31 (95% CI 0.28 to 0.34) for LSS cases, that is, the models missed on average by 11 and 12 ODI points. Calibration plots showed acceptable calibration (figure 2).

**Table 3:** Performance of XGBoost models

Model	Training		Test	
	MAE (95% CI)	R^2^ (95% CI)	MAE (95% CI)	R^2^ (95% CI)
LDH				
ODI	10.91 (10.77 to 11.04)	0.31 (0.27 to 0.32)	11.32 (11.00 to 11.63)	0.27 (0.24 to 0.29)
NRS leg pain	1.93 (1.91 to 1.95)	0.21 (0.20 to 0.22)	1.95 (1.90 to 2.00)	0.21 (0.19 to 0.23)
NRS back pain	2.00 (1.98 to 2.03)	0.17 (0.16 to 0.18)	2.09 (2.04 to 2.15)	0.17 (0.14 to 0.19)
LSS				
ODI	11.91 (11.78 to 12.04)	0.33 (0.32 to 0.35)	12.05 (11.76 to 12.32)	0.31 (0.28 to 0.34)
NRS leg pain	2.11 (2.09 to 2.13)	0.19 (0.18 to 0.20)	2.13 (2.08 to 2.16)	0.19 (0.17 to 0.21)
NRS back pain	2.33 (2.30 to 2.35)	0.15 (0.14 to 0.15)	2.33 (2.28 to 2.38)	0.12 (0.10 to 0.14)

LDH, lumbar disc herniation; LSS, lumbar spinal stenosis; MAE, mean absolute error; NRS, Numeric Rating Scale; ODI, Oswestry Disability Index; R2, coefficient of determination; XGBoost, extreme gradient boosting.

The models performed almost identically and well within the same CIs when trained with versus without imputation of cases with available 3 month but missing 12 month outcome (onlinesupplemental tables S6  S7).

#### Fairness

**Figure 3:**
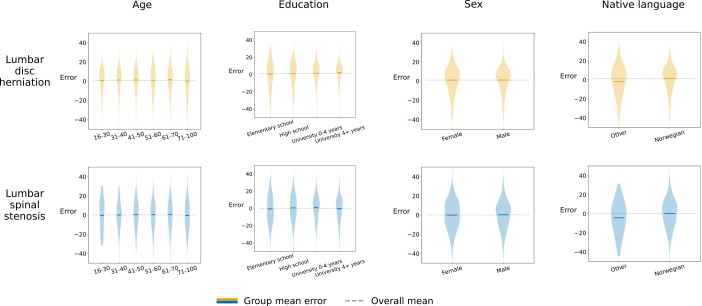
Oswestry Disability Index (ODI) model errors by age, education, sex and native language. The violin plots show the distribution of ODI error, grouped by age, education, sex and native language in the test set. Mean error (0.24) for the entire test set is shown in the grey dotted line. Group mean error is shown as a bold line within each violin.

Figure 3 shows the distribution and mean model error within demographic subgroups. There was almost no difference in error across sexes. Weak tendencies towards larger ranges in erroneous predictions of ODI were seen with increasing age and lower education. Distributions around the means were very similar. The models consistently underestimated improvement among non-native Norwegian speakers, with mean errors in the predicted ODI of −1.9 versus 1.3 (LDH) and −4.3 versus 0.3 (LSS).

#### Sensitivity analyses

The LDH and LSS regression models predicting ODI showed good and moderate discriminatory ability for classification of success, with AUCs of 0.82 (95% CI 0.81 to 0.84) and 0.72 (95% CI 0.70 to 0.73), respectively. The discriminatory ability had overlapping CIs to the logistic regression algorithm for both LDH (AUC 0.83, 95% CI 0.82 to 0.85) and LSS (AUC 0.70, 95% CI 0.68 to 0.71).

#### Patient similarity

The model found 50 comparators within the predetermined constraints for 97% of the test cases. online supplemental figure S1 in the supplementary files shows violin plots of normalised patient-similarity distances to the nearest cases for cases in the test set. The distributions and average distances were quite similar within each condition (LDH and LSS) and outcome (ODI, NRS back and leg pain). There were somewhat larger distances to similar patients for younger cases with LSS. Overall, the retrieval algorithms for both LDH and LSS showed consistent similarity (and thus performance) across age, education, native language and sex.

### Model updating

We conducted no updates to existing models.

## Discussion

This is, to our knowledge, the first study to develop AI-enabled models for the prediction of continuous outcome scores after lumbar spine surgery. The main findings were that the XGBoost regression models predicting ODI and NRS scores for disability and pain were accurate and well calibrated for both LDH and LSS. The MAEs were 11–12 points for ODI and around 2 points for prediction of NRS scores for leg and back pain. These errors are small and probably not important for patients. The models were fair with low variation across demographic subgroups for sex, age, education level and native language.

### Prediction of continuous outcomes

More specifically, the XGBoost models predicted ODI 12 months after operations for LDH and LSS with MAEs of 11.32 and 12.05 points, respectively. The MAEs for predictions of NRS scores for back and leg pain for LDH and LSS ranged from 1.95 to 2.33. These errors are slightly above the SEs of measurements of around 6 points for the ODI and 1 point for NRS.^41 42^ This is probably more accurate than surgeons’ discretionary predictions, and thus valuable for patients. The violin plots show, however, expected variations around the means, implying a risk for larger errors in individual cases.

There are few previous studies and no AI-enabled studies for direct comparison, since most published models are classifiers.^43 44^

The most relevant comparator is probably the study by McGirt *et al.*^45^ They studied 1803 consecutive cases that underwent various surgical procedures in the lumbar spine over a 4-year period in a single medical centre in the USA and used Bayesian model averaging to fit one linear regression model predicting ODI after 12 months. The model included numerous predictors similar to ours, and the authors reported high explanatory power with an R^2^ of 0.47. This compares favourably to the R^2^ of 0.27 for LDH and 0.31 for LSS in the present study. High explanatory power could indicate an accurate model but also overfitting in a model trained on a small and homogenous dataset. Contrary to McGirt *et al*,^45^ we used a much larger dataset from a national registry which captures the entire range of surgical practices and patients across many centres and a longer time span. More importantly, error is of greater significance than explanatory power in a clinical setting but McGirt *et al*^45^ did not report measures of error.

Two smaller studies also reported the prediction of ODI after lumbar spine surgery. Ford *et al* trained a linear regression model for the prediction of ODI after 6 months on data from 97 patients undergoing lumbar discectomy in a single centre in Australia and reported an R^2^ of 0.32.^46^ Alodaibi *et al*^47^ focused narrowly on fear avoidance for the prediction of ODI 10 weeks after surgery for LDH. They fitted a linear regression model trained on 60 patients and reported an R^2^ of 0.38.^47^ The small samples in these studies raise concerns about statistical power and overfitting and thus generalisability. Further, the absence of measures of error and the various and relatively short follow-up times limit clinical relevance and hamper comparison with our study.

#### Sensitivity analyses

In the classification setting, our models showed very good and good accuracy for the prediction of success after operations for LDH (AUC 0.82) and LSS (AUC 0.72), respectively. In the literature, the AUCs for most published models range between 0.6 and 0.9 with consistently higher values for LDH than LSS.^11^ Our findings align with the upper part of this range which confirms comparability with previous model development.

We did not reach higher AUCs than previous studies despite numerous predictor variables, a very large dataset and the use of advanced AI-enabled models. This highlights the shortcomings of classification, especially in clinical situations where the magnitude of an improvement is important, such as for disability or pain. Classifying outcomes as such also has shortcomings in situations where misclassifications are not considered equally disadvantageous, for example, when using a dichotomous outcome (success vs non-success) to select patients for surgical treatment.

AUC can be misleading when the size of the prediction error is important (such as for ODI) and when different classification errors are not viewed as equally problematic (such as when success/non-success predictions are used for selection for surgery). Issues of ‘one size fits all’ cut-offs in defining success have been highlighted in previous studies^48^ which argue against using rules of thumb for interpreting AUC values in our case.^49^ A core reason why we want to predict continuous outcomes is that the size and type of errors are weighted differently by different clinicians and patients. Further, by predicting continuous outcomes, we seem to have overcome some of the challenges in predicting outcomes for LSS cases. Published prediction models for LSS cases rarely discriminate better than AUC 0.70.^11 43^ Yet, for individual prediction with continuous outcomes, we show strong performance also for LSS cases.

#### Fairness

Our models were fair. Differences in performance were consistently below the SE of measurement across all sociodemographic subgroups which indicates the differences would be small in clinical practice.^41 42^ We hypothesise that clinical use may reduce differences in outcomes across subgroups such as native versus non-native speakers but this remains uncertain until tested in clinical trials.

#### Patient similarity

Evaluation of patient-similarity models is challenging. A scoping review reported that most studies used neighbourhood-based approaches. A knowledge gap was identified with regard to definitions of similarity metrics.^18^

We also used a neighbourhood-based approach (KNN) and developed a metric which ranks similarity with a weighted Manhattan distance. This is a new and innovative approach which performed equally across age, education, native language and sex with regard to distance from the case to previous similar cases retrieved by the model. No similar studies are available for comparison.

After discussion between computer scientists and surgeons, we discretionarily chose to retrieve outcomes for the 50 most similar cases to balance precision in similarity at baseline and a representative sample of outcomes. In a decision support tool, outcomes in this sample can be presented as mean scores or as proportions reaching cut-off-based predefined outcome categories (that is, complete recovery, substantial improvement, no significant change and worsening).

### Limitations

NORspine is a procedure-based registry which only records outcomes after surgical treatments. Therefore, our models do not consider outcomes of alternative treatments. The models are intended for use in Norwegian specialist healthcare in settings where spine surgeons assess patients who are referred for an evaluation of whether a scheduled operation should be provided or not. The models’ performance, safety and clinical effectiveness in the intended setting remain unknown until studied in clinical trials.

The models are not trained for use in situations where an acute operation is being considered. NORspine’s data are not representative for training such models because the capture rate for acute operations is low (34%). In addition, NORspine records reoperation within 90 days as treatment of a complication which means that we could not predict the outcomes of such reoperations separately. This implies that the models developed in this study cannot be used for decision-making in acute situations (eg, for patients with an acute paresis caused by an LDH) or for the treatment of complications.

In this study, we report lower proportions with successful outcomes than in earlier reports from NORspine.^28^ This is also because we excluded acute operations, which generally have better outcomes than elective procedures,^50^ and because we used stricter cut-offs for success than previously (22 vs 20 ODI points for LDH and 14 ODI points vs a 30% improvement in ODI for LSS) to incentivise quality improvement.

Our prediction models rely heavily on patients’ subjective assessments, which could cause self-reporting bias, since most variables are derived from patients’ questionnaires. NORspine has documented high validity and reliability of the patient-reported data. In 2022, >80% responded at 3-month and 12-month follow-up, and response completeness for key single questions was 92%–100%.^4^ The variables used in our models had a completeness of more than 90%.

The models achieved moderate explanatory power, with an R^2^ of 0.28 for LDH and 0.31 for LSS, despite the use of numerous predictor variables. This indicates a risk for unmeasured confounding and a potential for improved predictions if new predictors could be identified.

#### Limitations related to model architecture

XGBoost is a supervised ensemble machine learning method based on aggregating multiple decision trees. It is well established and known to perform well in structured data prediction benchmarks.^51 52^ However, like other decision-tree-based algorithms, it requires hyperparameter tuning to avoid overfitting.

### Usability of the model in the context of current care

A recent review reported that all AI-enabled models for outcome prediction after spine surgery are stand-alone or web-based systems. None have been implemented in routine clinical practice or integrated into an electronic health record (EHR).^44^ The Swedish spine registry used multivariable logistic regression to develop Dialogue Support, a web-based patient-specific prediction tool that uses 15 baseline variables to predict outcomes and plans to test its efficacy in a multicentre clinical trial.^53^ We argue that single-password access in physicians’ regular user interfaces is a prerequisite for successful implementation and therefore, aim to integrate NORspine’s questionnaires and decision support based on the prediction models reported in this study in the two EHRs used in Norway.

## Future work

Our results show that we have made a promising basis for developing a decision support tool for the selection of patients for spine surgery. The process of designing and integrating the decision support into the EHR is ongoing and will be reported separately. The initial feasibility studies, which include iterative development and testing of user interfaces with graphical display of the predictions and clinical trials that evaluate whether use of the models for decision support can increase the proportion of patients who achieve substantial improvement, have been approved by the Norwegian Medical Products Agency (CIV-NO-24-06-047736) and are registered on clinicaltrials.gov (NCT06806969).

## Supplementary material

10.1136/bmjopen-2025-108947 online supplemental file 1

10.1136/bmjopen-2025-108947 online supplemental file 2

10.1136/bmjopen-2025-108947 online supplemental file 3

10.1136/bmjopen-2025-108947 online supplemental file 4

10.1136/bmjopen-2025-108947 online supplemental file 5

10.1136/bmjopen-2025-108947 online supplemental file 6

10.1136/bmjopen-2025-108947 online supplemental file 7

10.1136/bmjopen-2025-108947 online supplemental file 8

## Data Availability

Data are available upon reasonable request.
