# Supplementary material for: Prediction of Oswestry Disability Index and Numeric Rating Scale scores after lumbar spine surgery: machine learning model development and fairness assessment
Source: BMJ Open. 2026 May 13;16(5):e108947. doi: 10.1136/bmjopen-2025-108947 (PMC13182469; doi:10.1136/bmjopen-2025-108947)
Supplement: online supplemental file 1 [file bmjopen-16-5-s001.docx]

# Table S1 – Presurgical variables registered by NORspine

Variables are listed with applicable options in parentheses. The following variables are collected from questionnaires issued to the patients:

- Whether patient smokes (yes/no/previously)
- Whether patient uses snuff/snus (yes/no/previously)
- Patient height (m)
- Patient weight (kg)
- Any prior treatment for current problems (yes/no)
- Effect of prior treatments (individual questions, answered with improvement/no change/worsening/not applicable)
  - Effect of training with physiotherapist
  - Effect of other treatment from physiotherapist
  - Effect of manual therapy
  - Effect of psychomotor physiotherapy
  - Effect of chiropractic treatment
  - Effect of treatment at multidisciplinary outpatient clinic for back patients, or treatment at rehabilitation centre
  - Effect of other treatment
- Duration of patient’s back/hip pain (no back/hip pain, less than 3 months, 3-12 months, 1-2 years, more than 2 years)
- Duration of patient’s leg pain (no radiating pain, less than 3 months, 3-12 months, 1-2 years, more than 2 years)
- Waiting time from referral from patient’s GP until completed appointment at specialist outpatient clinic (less than 3 months, 3-6 months, 6-12 months, more than 1 year)
- Waiting time from decision to perform surgery until completed operation (less than 3 months, 3-6 months, 6-12 months, more than 1 year)
- Whether patient uses painkillers due to back and/or leg pain (yes/no)
- How often the patient uses painkillers (not applicable, less often than monthly, every month, every week, daily, several times a day)
- Patient-reported severity of back/hip pain in the past week (0-10)
- Patient-reported severity of leg pain in the past week (0-10)
- Patient answers to the Oswestry Low Back Pain Disability Questionnaire (individual questions answered on a 6-point scale, ranging from 0 or no impact on the category in question, to 5 or complete impairment from back/leg pain)
  - Pain intensity
  - Personal care
  - Lifting
  - Walking
  - Sitting
  - Standing
  - Sleeping
  - Sex life (if applicable)
  - Social life
  - Travelling
- Patient’s Oswestry Disability Index, calculated from the Oswestry Low Back Pain Disability Questionnaire
- Patient answers to the EQ-5D-5L Health Questionnaire (answered on a 5-point scale, ranging from 1 or no problems in the given category, to 5 or complete impairment from back/leg pain)
  - Mobility
  - Self-care
  - Usual activities
  - Pain/discomfort
  - Anxiety/depression
- Patient’s EQ-5D index, calculated from the EQ-5D-5L Questionnaire and the UK value set
- Patient’s self-assessed state of health (scored on VAS scale from 0 to 100)
- Patient’s belief that current pain will become persistent (answered on 0-10 scale)
- Patient’s belief that they will resume their current work in 6 months (answered on 0-10 scale)
- Patient’s belief that increased pain indicates they should stop activity until the pain subsides (answered on 0-10 scale)
- Patient’s belief that their current pain means they should not do their current work (answered on 0-10 scale)
- Patient’s marital status (married or cohabiting/single)
- Whether patient has difficulties reading or writing (none/to some extent/a great deal)
- Patient’s employment status before surgery (employed full time, employed part time, student/pupil, retired, unemployed, on sick leave, on partial sick leave, on work assessment allowance, receiving disability benefits)
- Duration of sick leave for back-related problems (not applicable, less than 3 months, 3-6 months, 6-12 months, more than 1 year)
- Patient’s belief that their current employer would like to have them back in the workplace (yes/no/do not know)
- Whether patient has applied for a disability pension (yes/no/planning to/pension already granted)
- Whether patient has applied for compensation from any insurance company, including the Norwegian patient injury compensation scheme or occupational injury compensation (yes/no/planning to/compensation already granted)
- Patient’s assessment of their work as physically heavy (scored on a 0-10 scale)
- Patient’s assessment of their work as monotonous (scored on a 0-10 scale)
- Patient’s first language (Norwegian/Sami/other, specified in the form)
- Patient’s level of education (primary school, vocational school, upper secondary, less than 4 years of university, 4 years of university or more)
- Patient’s ethnic/cultural affiliation

The following variables are collected from surgeons’ registration forms:

- Whether patient has undergone spine surgery previously (no/yes, same vertebra/yes, different vertebra/yes, on same and different vertebra/unknown)
- Whether patient is taking blood thinning medication regularly (yes, with the medication and date of discontinuation specified/no)
- Whether patient is taking steroids (yes/no)
- Whether patient is on other immunosuppressive treatment (yes/no)
- Whether patient is suffering from other relevant conditions: (individual yes/no questions, unless otherwise specified)
  - Rheumatoid arthritis
  - Ankylosing spondylitis
  - Other rheumatic disease
  - Hip- or knee arthrosis
  - Depression/anxiety
  - Generalised pain syndrome
  - Chronic neurological disease
  - Cerebrovascular disease
  - Cardiovascular disease
  - Polyneuropathy
  - Vascular claudication
  - Chronic pulmonary disease
  - Cancer
  - Osteoporosis
  - Osteoporotic thoracolumbar fracture
  - Hypertension
  - Diabetes mellitus
  - Other endocrine disorders
  - Prostatism
  - Other relevant conditions (free text)
- Whether a CT examination has been conducted (yes/no)
- Whether an MRI examination has been conducted (yes/no)
- Whether X-ray imaging of the lumbosacral spine has been conducted (yes/no)
- Whether a diagnostic blockade has been performed (yes/yes, in facet joint/yes in nerve root/yes, in facet joint and nerve root/no)
- Clinical findings during the above examinations (individual yes/no questions, unless otherwise specified)
  - Prolapse
  - Intraforaminal prolapse
  - Extreme lateral/extraforaminal prolapse
  - Central spinal stenosis
  - Lateral/recess stenosis
  - Foraminal stenosis
  - Only disc degeneration/spondylosis without nerve affection
  - Isthmic spondylolysis
  - Isthmic spondylolisthesis (yes, with Meyerding grade specified on an I-IV scale/no)
  - Degenerative spondylolisthesis observed from MRI (yes, with no. of millimetres of displacement specified/no)
  - Degenerative scoliosis
  - Kyphosis
  - Synovial cyst
- Whether patient is experiencing paresis (yes, with degree of paresis specified on a 0-5 scale/no)
- Duration of paresis (none/less than 24 hours/24 hours-1 week/1 week-3 months/more than 3 months)
- Whether patient has cauda equina syndrome (yes/no)
- Duration of cauda equina syndrome (none/less than 24 hours/24 hours-1 week/1 week-3 months/more than 3 months)
