# Supplementary material for: Prediction of Oswestry Disability Index and Numeric Rating Scale scores after lumbar spine surgery: machine learning model development and fairness assessment
Source: BMJ Open. 2026 May 13;16(5):e108947. doi: 10.1136/bmjopen-2025-108947 (PMC13182469; doi:10.1136/bmjopen-2025-108947)
Supplement: online supplemental file 2 [file bmjopen-16-5-s002.docx]

Table S2 Extended baseline characteristics of cases included in the ODI models development

| Variables at baseline | LDH | | | LSS | | |
| --- | --- | --- | --- | --- | --- | --- |
|  | Included (n=18377) | | Excluded cases due to no follow-up ODI (n=4753) | Included (n=24540) | | Excluded cases due to no follow-up ODI (n=3579) |
|  | Cases with available 12-month ODI (n=15398) | Cases with available 3-month, but not 12-month ODI (n=2979) |  | Cases with available 12-month ODI (n=21640) | Cases with available 3-month, but not 12-month ODI (n=2900) |  |
| Sex, n |  |  |  |  |  |  |
| *Missing* | *0 (0.0%)* | *0 (0.0%)* | *0 (0.0%)* | *0 (0.0%)* | *0 (0.0%)* | *0 (0.0%)* |
| Female | 6629 (43.1%) | 1194 (40.1%) | 1669 (35.1%) | 11059 (51.1%) | 1504 (51.9%) | 1759 (49.1%) |
| Male | 8769 (56.9%) | 1785 (59.9%) | 3084 (64.9%) | 10581 (48.9%) | 1396 (48.1%) | 1820 (50.9%) |
| Age, mean (SD) | 49.09 (14.40) | 44.44 (13.54) | 41.52 (12.70) | 66.28 (11.29) | 63.86 (13.14) | 61.01 (13.67) |
| *Missing, n* | *41 (0.3%)* | *15 (0.5%)* | *22 (0.5%)* | *33 (0.2%)* | *2 (0.1%)* | *11 (0.3%)* |
| Smoking status, n |  |  |  |  |  |  |
| *Missing* | *131 (0.9%)* | *24 (0.8%)* | *51 (1.1%)* | *205 (0.9%)* | *31 (1.1%)* | *44 (1.2%)* |
| Yes | 3283 (21.3%) | 846 (28.4%) | 1426 (30.0%) | 3690 (17.1%) | 646 (22.3%) | 982 (27.4%) |
| No | 11984 (77.8%) | 2109 (70.8%) | 3276 (68.9%) | 17745 (82.0%) | 2223 (76.7%) | 2553 (71.3%) |
| BMI, mean (SD) | 26.87 (4.33) | 27.09 (4.51) | 27.21 (4.53) | 27.68 (4.41) | 27.69 (4.61) | 27.99 (4.71) |
| *Missing, n (%)* | *864 (5.6%)* | *175 (5.9%)* | *251 (5.3%)* | *891 (4.1%)* | *124 (4.3%)* | *166 (4.6%)* |
| Symptom duration (back or hip pain), n |  |  |  |  |  |  |
| *Missing* | *497 (3.2%)* | *95 (3.2%)* | *181 (3.8%)* | *970 (4.5%)* | *132 (4.6%)* | *162 (4.5%)* |
| No symptoms | 0 (0.0%) | 0 (0.0%) | 0 (0.0%) | 0 (0.0%) | 0 (0.0%) | 0 (0.0%) |
| 0 to 3 months | 417 (2.7%) | 55 (1.8%) | 98 (2.1%) | 441 (2.0%) | 55 (1.9%) | 57 (1.6%) |
| 3 to 12 months | 1597 (10.4%) | 276 (9.3%) | 460 (9.7%) | 446 (2.1%) | 56 (1.9%) | 79 (2.2%) |
| 12 to 24 months | 7052 (45.8%) | 1372 (46.1%) | 2135 (44.9%) | 4649 (21.5%) | 593 (20.4%) | 726 (20.3%) |
| More than 24 months | 2407 (15.6%) | 493 (16.5%) | 772 (16.2%) | 4504 (20.8%) | 582 (20.1%) | 762 (21.3%) |
| Symptom duration (leg pain) , n |  |  |  |  |  |  |
| *Missing* | *620 (4.0%)* | *126 (4.2%)* | *222 (4.7%)* | *1314 (6.1%)* | *196 (6.8%)* | *230 (6.4%)* |
| No symptoms | 0 (0.0%) | 0 (0.0%) | 0 (0.0%) | 0 (0.0%) | 0 (0.0%) | 0 (0.0%) |
| 0 to 3 months | 212 (1.4%) | 33 (1.1%) | 71 (1.5%) | 704 (3.3%) | 78 (2.7%) | 95 (2.7%) |
| 3 to 12 months | 2428 (15.8%) | 423 (14.2%) | 671 (14.1%) | 775 (3.6%) | 108 (3.7%) | 143 (4.0%) |
| 12 to 24 months | 8068 (52.4%) | 1560 (52.4%) | 2435 (51.2%) | 6273 (29.0%) | 781 (26.9%) | 961 (26.9%) |
| More than 24 months | 2138 (13.9%) | 446 (15.0%) | 699 (14.7%) | 5154 (23.8%) | 703 (24.2%) | 833 (23.3%) |
| Use of painkillers, n |  |  |  |  |  |  |
| *Missing* | *59 (0.4%)* | *12 (0.4%)* | *26 (0.5%)* | *147 (0.7%)* | *10 (0.3%)* | *33 (0.9%)* |
| No | 2379 (15.5%) | 434 (14.6%) | 722 (15.2%) | 4239 (19.6%) | 511 (17.6%) | 643 (18.0%) |
| Yes | 12960 (84.2%) | 2533 (85.0%) | 4005 (84.3%) | 17254 (79.7%) | 2379 (82.0%) | 2903 (81.1%) |
| NRS back pain, mean (SD) | 6.12 (2.36) | 6.23 (2.33) | 6.19 (2.29) | 6.52 (2.17) | 6.69 (2.15) | 6.64 (2.18) |
| *Missing, n* | *424 (2.8%)* | *69 (2.3%)* | *166 (3.5%)* | *1356 (6.3%)* | *231 (8.0%)* | *252 (7.0%)* |
| NRS leg pain, mean (SD) | 6.73 (2.15) | 6.75 (2.14) | 6.67 (2.13) | 6.56 (2.22) | 6.76 (2.19) | 6.61 (2.25) |
| *Missing, n* | *410 (2.7%)* | *79 (2.7%)* | *167 (3.5%)* | *1573 (7.3%)* | *274 (9.4%)* | *289 (8.1%)* |
| ODI, mean (SD) | 42.78 (16.71) | 42.95 (17.11) | 42.57 (16.90) | 38.61 (14.91) | 40.71 (15.15) | 41.51 (15.68) |
| *Missing, n* | *0 (0.0%)* | *0 (0.0%)* | *0 (0.0%)* | *0 (0.0%)* | *0 (0.0%)* | *0 (0.0%)* |
| EQ5D index, mean (SD) | 0.36 (0.32) | 0.34 (0.32) | 0.35 (0.32) | 0.43 (0.29) | 0.39 (0.30) | 0.37 (0.30) |
| *Missing, n* | *491 (3.2%)* | *105 (3.5%)* | *188 (4.0%)* | *1157 (5.3%)* | *169 (5.8%)* | *251 (7.0%)* |
| EQ5D anxiety score, n |  |  |  |  |  |  |
| *Missing* | *215 (1.4%)* | *38 (1.3%)* | *98 (2.1%)* | *527 (2.4%)* | *83 (2.9%)* | *117 (3.3%)* |
| Not anxious or depressed (level 1) | 8602 (55.9%) | 1549 (52.0%) | 2482 (52.2%) | 12729 (58.8%) | 1532 (52.8%) | 1756 (49.1%) |
| Slightly anxious or depressed (level 2) | 1491 (9.7%) | 271 (9.1%) | 438 (9.2%) | 2440 (11.3%) | 346 (11.9%) | 361 (10.1%) |
| Moderately anxious or depressed (level 3) | 4528 (29.4%) | 971 (32.6%) | 1497 (31.5%) | 5289 (24.4%) | 804 (27.7%) | 1138 (31.8%) |
| Severely anxious or depressed (level 4) | 177 (1.1%) | 46 (1.5%) | 87 (1.8%) | 309 (1.4%) | 55 (1.9%) | 77 (2.2%) |
| Extremely anxious or depressed (level 5) | 385 (2.5%) | 104 (3.5%) | 151 (3.2%) | 346 (1.6%) | 80 (2.8%) | 130 (3.6%) |
| Civil status, n |  |  |  |  |  |  |
| *Missing* | *136 (0.9%)* | *23 (0.8%)* | *54 (1.1%)* | *202 (0.9%)* | *37 (1.3%)* | *43 (1.2%)* |
| Living alone | 3529 (22.9%) | 819 (27.5%) | 1441 (30.3%) | 5467 (25.3%) | 840 (29.0%) | 1177 (32.9%) |
| Cohabiting | 11733 (76.2%) | 2137 (71.7%) | 3258 (68.5%) | 15971 (73.8%) | 2023 (69.8%) | 2359 (65.9%) |
| Work status, n |  |  |  |  |  |  |
| *Missing* | *515 (3.3%)* | *78 (2.6%)* | *197 (4.1%)* | *1107 (5.1%)* | *138 (4.8%)* | *202 (5.6%)* |
| Working or student | 4171 (27.1%) | 784 (26.3%) | 1335 (28.1%) | 3453 (16.0%) | 516 (17.8%) | 627 (17.5%) |
| Retired | 1886 (12.2%) | 199 (6.7%) | 186 (3.9%) | 10551 (48.8%) | 1180 (40.7%) | 1180 (33.0%) |
| Sick leave | 6732 (43.7%) | 1496 (50.2%) | 2275 (47.9%) | 3027 (14.0%) | 476 (16.4%) | 765 (21.4%) |
| Unemployed / Work settlement allowance / Disability pension | 2094 (13.6%) | 422 (14.2%) | 760 (16.0%) | 3502 (16.2%) | 590 (20.3%) | 805 (22.5%) |
| Education, n |  |  |  |  |  |  |
| *Missing* | *186 (1.2%)* | *32 (1.1%)* | *81 (1.7%)* | *708 (3.3%)* | *114 (3.9%)* | *144 (4.0%)* |
| Elementary school, 7-10 years | 2122 (13.8%) | 421 (14.1%) | 671 (14.1%) | 4737 (21.9%) | 702 (24.2%) | 827 (23.1%) |
| High school | 6950 (45.1%) | 1450 (48.7%) | 2398 (50.5%) | 9027 (41.7%) | 1204 (41.5%) | 1597 (44.6%) |
| University <4 years | 3209 (20.8%) | 584 (19.6%) | 906 (19.1%) | 3882 (17.9%) | 470 (16.2%) | 563 (15.7%) |
| University >5 years | 2931 (19.0%) | 492 (16.5%) | 697 (14.7%) | 3286 (15.2%) | 410 (14.1%) | 448 (12.5%) |
| Applied for disability benefits, n |  |  |  |  |  |  |
| *Missing* | *1065 (6.9%)* | *171 (5.7%)* | *278 (5.8%)* | *5043 (23.3%)* | *660 (22.8%)* | *667 (18.6%)* |
| No | 12592 (81.8%) | 2496 (83.8%) | 3964 (83.4%) | 12959 (59.9%) | 1680 (57.9%) | 2228 (62.3%) |
| Planning to | 232 (1.5%) | 51 (1.7%) | 88 (1.9%) | 322 (1.5%) | 38 (1.3%) | 61 (1.7%) |
| Yes | 242 (1.6%) | 59 (2.0%) | 86 (1.8%) | 419 (1.9%) | 65 (2.2%) | 86 (2.4%) |
| Already approved | 1267 (8.2%) | 202 (6.8%) | 337 (7.1%) | 2897 (13.4%) | 457 (15.8%) | 537 (15.0%) |
| Previously operated, n |  |  |  |  |  |  |
| *Missing* | *80 (0.5%)* | *23 (0.8%)* | *25 (0.5%)* | *144 (0.7%)* | *24 (0.8%)* | *33 (0.9%)* |
| Yes | 3213 (20.9%) | 682 (22.9%) | 1178 (24.8%) | 5300 (24.5%) | 784 (27.0%) | 1007 (28.1%) |
| No | 12105 (78.6%) | 2274 (76.3%) | 3550 (74.7%) | 16196 (74.8%) | 2092 (72.1%) | 2539 (70.9%) |
| Number of previous operations, mean (SD) | 0.25 (0.58) | 0.28 (0.62) | 0.31 (0.65) | 0.32 (0.68) | 0.35 (0.69) | 0.38 (0.73) |
| *Missing, n* | *379 (2.5%)* | *96 (3.2%)* | *134 (2.8%)* | *635 (2.9%)* | *83 (2.9%)* | *115 (3.2%)* |
| Has other relevant diseases, n |  |  |  |  |  |  |
| *Missing* | *1120 (7.3%)* | *249 (8.4%)* | *333 (7.0%)* | *1120 (5.2%)* | *132 (4.6%)* | *199 (5.6%)* |
| Yes | 5141 (33.4%) | 900 (30.2%) | 1342 (28.2%) | 14119 (65.2%) | 1875 (64.7%) | 2173 (60.7%) |
| No | 9137 (59.3%) | 1830 (61.4%) | 3078 (64.8%) | 6401 (29.6%) | 893 (30.8%) | 1207 (33.7%) |
| Degree of paresis, n |  |  |  |  |  |  |
| *Missing* | *0 (0.0%)* | *0 (0.0%)* | *0 (0.0%)* | *0 (0.0%)* | *0 (0.0%)* | *0 (0.0%)* |
| Total paralysis (0) | 48 (0.3%) | 10 (0.3%) | 12 (0.3%) | 83 (0.4%) | 10 (0.3%) | 15 (0.4%) |
| Palpable or visible contraction (1) | 42 (0.3%) | 2 (0.1%) | 6 (0.1%) | 44 (0.2%) | 5 (0.2%) | 8 (0.2%) |
| Active movement, gravity eliminated (2) | 94 (0.6%) | 10 (0.3%) | 14 (0.3%) | 64 (0.3%) | 10 (0.3%) | 10 (0.3%) |
| Active movement, against gravity (3) | 431 (2.8%) | 72 (2.4%) | 98 (2.1%) | 211 (1.0%) | 32 (1.1%) | 43 (1.2%) |
| Active movement, against some resistance (4) | 1356 (8.8%) | 276 (9.3%) | 386 (8.1%) | 835 (3.9%) | 117 (4.0%) | 147 (4.1%) |
| Active movement, against full resistance (5) | 329 (2.1%) | 56 (1.9%) | 103 (2.2%) | 352 (1.6%) | 46 (1.6%) | 50 (1.4%) |
| No symptoms (6) | 13098 (85.1%) | 2553 (85.7%) | 4134 (87.0%) | 20051 (92.7%) | 2680 (92.4%) | 3306 (92.4%) |
| ASA score, n |  |  |  |  |  |  |
| *Missing* | *195 (1.3%)* | *41 (1.4%)* | *57 (1.2%)* | *312 (1.4%)* | *42 (1.4%)* | *56 (1.6%)* |
| Normal health patient (1) | 6934 (45.0%) | 1409 (47.3%) | 2304 (48.5%) | 2880 (13.3%) | 447 (15.4%) | 603 (16.8%) |
| Patient with mild systemic disease (2) | 7158 (46.5%) | 1352 (45.4%) | 2170 (45.7%) | 13539 (62.6%) | 1700 (58.6%) | 2164 (60.5%) |
| Patient with severe systemic disease (3) | 1097 (7.1%) | 174 (5.8%) | 216 (4.5%) | 4838 (22.4%) | 701 (24.2%) | 746 (20.8%) |
| Patient with severe systemic disease that  is a constant threat to life (4) | 13 (0.1%) | 3 (0.1%) | 6 (0.1%) | 67 (0.3%) | 10 (0.3%) | 10 (0.3%) |
| Moribund patient not expected to survive  without the operation (5) | 1 (0.0%) | 0 (0.0%) | 0 (0.0%) | 4 (0.0%) | 0 (0.0%) | 0 (0.0%) |
