# Supplementary material for: Prediction of Oswestry Disability Index and Numeric Rating Scale scores after lumbar spine surgery: machine learning model development and fairness assessment
Source: BMJ Open. 2026 May 13;16(5):e108947. doi: 10.1136/bmjopen-2025-108947 (PMC13182469; doi:10.1136/bmjopen-2025-108947)
Supplement: online supplemental file 3 [file bmjopen-16-5-s003.docx]

# Table S3 Baseline characteristics of cases used for training NRS back pain models

| Variables at baseline | LDH | | | LSS | | |
| --- | --- | --- | --- | --- | --- | --- |
|  | Included (n=17913) | | Excluded due to missing follow-up (n=4612) | Included (n=23089) | | Included (n=23089)  Cases with available 12-month outcome (n=20377) |
|  | Cases with available 12-month outcome (n=14983) | Cases with available 3-month outcome, but not 12-month outcome (n=2930) |  | Cases with available 12-month outcome (n=20377) | Cases with available 3-month outcome, but not 12-month outcome (n=2712) |  |
| Sex, percent female |  |  |  |  |  |  |
| *Missing, n (%)* | *0 (0.0%)* | *0 (0.0%)* | *0 (0.0%)* | *0 (0.0%)* | *0 (0.0%)* | *0 (0.0%)* |
| Female | 6420 (42.8%) | 1171 (40.0%) | 1615 (35.0%) | 10335 (50.7%) | 1395 (51.4%) | 1673 (49.4%) |
| Male | 8563 (57.2%) | 1759 (60.0%) | 2997 (65.0%) | 10042 (49.3%) | 1317 (48.6%) | 1711 (50.6%) |
| Age, mean (SD) | 48.82 (14.29) | 44.30 (13.55) | 41.36 (12.56) | 65.92 (11.30) | 63.44 (13.09) | 60.60 (13.68) |
| *Missing, n (%)* | *41 (0.3%)* | *15 (0.5%)* | *22 (0.5%)* | *30 (0.1%)* | *3 (0.1%)* | *10 (0.3%)* |
| Smoking status, percent |  |  |  |  |  |  |
| *Missing, n (%)* | *125 (0.8%)* | *24 (0.8%)* | *52 (1.1%)* | *194 (1.0%)* | *23 (0.8%)* | *41 (1.2%)* |
| Yes | 3180 (21.2%) | 827 (28.2%) | 1375 (29.8%) | 3490 (17.1%) | 612 (22.6%) | 934 (27.6%) |
| No | 11678 (77.9%) | 2079 (71.0%) | 3185 (69.1%) | 16693 (81.9%) | 2077 (76.6%) | 2409 (71.2%) |
| BMI, mean (SD) | 26.90 (4.33) | 27.09 (4.51) | 27.22 (4.52) | 27.72 (4.41) | 27.77 (4.60) | 28.02 (4.71) |
| *Missing, n (%)* | *838 (5.6%)* | *168 (5.7%)* | *243 (5.3%)* | *837 (4.1%)* | *122 (4.5%)* | *152 (4.5%)* |
| Symptom duration (back or hip pain), percent |  |  |  |  |  |  |
| *Missing, n (%)* | *437 (2.9%)* | *87 (3.0%)* | *158 (3.4%)* | *773 (3.8%)* | *102 (3.8%)* | *127 (3.8%)* |
| No symptoms | 0 (0.0%) | 0 (0.0%) | 0 (0.0%) | 0 (0.0%) | 0 (0.0%) | 0 (0.0%) |
| 0 to 3 months | 391 (2.6%) | 54 (1.8%) | 87 (1.9%) | 375 (1.8%) | 43 (1.6%) | 51 (1.5%) |
| 3 to 12 months | 1568 (10.5%) | 272 (9.3%) | 445 (9.6%) | 427 (2.1%) | 54 (2.0%) | 74 (2.2%) |
| 12 to 24 months | 6878 (45.9%) | 1349 (46.0%) | 2097 (45.5%) | 4427 (21.7%) | 560 (20.6%) | 686 (20.3%) |
| More than 24 months | 2368 (15.8%) | 491 (16.8%) | 759 (16.5%) | 4252 (20.9%) | 550 (20.3%) | 736 (21.7%) |
| Symptom duration (leg pain), percent |  |  |  |  |  |  |
| *Missing, n (%)* | *561 (3.7%)* | *117 (4.0%)* | *206 (4.5%)* | *1113 (5.5%)* | *163 (6.0%)* | *198 (5.9%)* |
| No symptoms | 0 (0.0%) | 0 (0.0%) | 0 (0.0%) | 0 (0.0%) | 0 (0.0%) | 0 (0.0%) |
| 0 to 3 months | 200 (1.3%) | 34 (1.2%) | 70 (1.5%) | 661 (3.2%) | 78 (2.9%) | 89 (2.6%) |
| 3 to 12 months | 2383 (15.9%) | 415 (14.2%) | 649 (14.1%) | 739 (3.6%) | 102 (3.8%) | 129 (3.8%) |
| 12 to 24 months | 7865 (52.5%) | 1540 (52.6%) | 2382 (51.6%) | 5949 (29.2%) | 736 (27.1%) | 911 (26.9%) |
| More than 24 months | 2093 (14.0%) | 449 (15.3%) | 680 (14.7%) | 4876 (23.9%) | 665 (24.5%) | 803 (23.7%) |
| Use of painkillers, percent |  |  |  |  |  |  |
| *Missing, n (%)* | *53 (0.4%)* | *10 (0.3%)* | *27 (0.6%)* | *123 (0.6%)* | *13 (0.5%)* | *22 (0.7%)* |
| No | 2301 (15.4%) | 425 (14.5%) | 705 (15.3%) | 3915 (19.2%) | 464 (17.1%) | 600 (17.7%) |
| Yes | 12629 (84.3%) | 2495 (85.2%) | 3880 (84.1%) | 16339 (80.2%) | 2235 (82.4%) | 2762 (81.6%) |
| NRS back pain, mean (SD) | 6.12 (2.36) | 6.23 (2.33) | 6.19 (2.29) | 6.52 (2.17) | 6.68 (2.17) | 6.65 (2.18) |
| *Missing, n (%)* | *0 (0.0%)* | *0 (0.0%)* | *0 (0.0%)* | *0 (0.0%)* | *0 (0.0%)* | *0 (0.0%)* |
| NRS leg pain, mean (SD) | 6.72 (2.15) | 6.74 (2.14) | 6.67 (2.13) | 6.55 (2.22) | 6.75 (2.21) | 6.60 (2.25) |
| *Missing, n (%)* | *137 (0.9%)* | *31 (1.1%)* | *46 (1.0%)* | *595 (2.9%)* | *102 (3.8%)* | *87 (2.6%)* |
| ODI, mean (SD) | 42.85 (16.71) | 42.94 (17.11) | 42.62 (16.88) | 38.77 (14.90) | 40.87 (15.04) | 41.59 (15.62) |
| *Missing, n (%)* | *31 (0.2%)* | *4 (0.1%)* | *19 (0.4%)* | *133 (0.7%)* | *29 (1.1%)* | *31 (0.9%)* |
| EQ5D index, mean (SD) | 0.36 (0.32) | 0.34 (0.32) | 0.35 (0.32) | 0.43 (0.29) | 0.39 (0.30) | 0.37 (0.30) |
| *Missing, n (%)* | *450 (3.0%)* | *102 (3.5%)* | *173 (3.8%)* | *997 (4.9%)* | *148 (5.5%)* | *203 (6.0%)* |
| EQ5D anxiety score, percent |  |  |  |  |  |  |
| *Missing, n (%)* | *193 (1.3%)* | *40 (1.4%)* | *93 (2.0%)* | *466 (2.3%)* | *65 (2.4%)* | *92 (2.7%)* |
| Not anxious or depressed (level 1) | 8367 (55.8%) | 1520 (51.9%) | 2407 (52.2%) | 11983 (58.8%) | 1436 (52.9%) | 1651 (48.8%) |
| Slightly anxious or depressed (level 2) | 1453 (9.7%) | 263 (9.0%) | 415 (9.0%) | 2292 (11.2%) | 308 (11.4%) | 332 (9.8%) |
| Moderately anxious or depressed (level 3) | 4426 (29.5%) | 961 (32.8%) | 1466 (31.8%) | 5018 (24.6%) | 769 (28.4%) | 1115 (32.9%) |
| Severely anxious or depressed (level 4) | 167 (1.1%) | 45 (1.5%) | 84 (1.8%) | 289 (1.4%) | 55 (2.0%) | 69 (2.0%) |
| Extremely anxious or depressed (level 5) | 377 (2.5%) | 101 (3.4%) | 147 (3.2%) | 329 (1.6%) | 79 (2.9%) | 125 (3.7%) |
| Civil status, percent |  |  |  |  |  |  |
| *Missing, n (%)* | *133 (0.9%)* | *21 (0.7%)* | *53 (1.1%)* | *180 (0.9%)* | *31 (1.1%)* | *38 (1.1%)* |
| Living alone | 3435 (22.9%) | 797 (27.2%) | 1398 (30.3%) | 5092 (25.0%) | 783 (28.9%) | 1102 (32.6%) |
| Cohabiting | 11415 (76.2%) | 2112 (72.1%) | 3161 (68.5%) | 15105 (74.1%) | 1898 (70.0%) | 2244 (66.3%) |
| Work status, percent |  |  |  |  |  |  |
| *Missing, n (%)* | *477 (3.2%)* | *75 (2.6%)* | *185 (4.0%)* | *979 (4.8%)* | *108 (4.0%)* | *189 (5.6%)* |
| Working or student | 4085 (27.3%) | 774 (26.4%) | 1307 (28.3%) | 3342 (16.4%) | 482 (17.8%) | 605 (17.9%) |
| Retired | 1769 (11.8%) | 192 (6.6%) | 172 (3.7%) | 9742 (47.8%) | 1087 (40.1%) | 1092 (32.3%) |
| Sick leave | 6602 (44.1%) | 1471 (50.2%) | 2222 (48.2%) | 2952 (14.5%) | 461 (17.0%) | 743 (22.0%) |
| Unemployed / Work settlement allowance / Disability pension | 2050 (13.7%) | 418 (14.3%) | 726 (15.7%) | 3362 (16.5%) | 574 (21.2%) | 755 (22.3%) |
| Education, percent |  |  |  |  |  |  |
| *Missing, n (%)* | *167 (1.1%)* | *31 (1.1%)* | *76 (1.6%)* | *612 (3.0%)* | *90 (3.3%)* | *123 (3.6%)* |
| Elementary school, 7-10 years | 2015 (13.4%) | 413 (14.1%) | 652 (14.1%) | 4319 (21.2%) | 630 (23.2%) | 787 (23.3%) |
| High school | 6771 (45.2%) | 1418 (48.4%) | 2310 (50.1%) | 8573 (42.1%) | 1124 (41.4%) | 1512 (44.7%) |
| University <4 years | 3150 (21.0%) | 582 (19.9%) | 884 (19.2%) | 3704 (18.2%) | 468 (17.3%) | 532 (15.7%) |
| University >5 years | 2880 (19.2%) | 486 (16.6%) | 690 (15.0%) | 3169 (15.6%) | 400 (14.7%) | 430 (12.7%) |
| Applied for disability benefits, percent |  |  |  |  |  |  |
| *Missing, n (%)* | *983 (6.6%)* | *167 (5.7%)* | *249 (5.4%)* | *4585 (22.5%)* | *573 (21.1%)* | *588 (17.4%)* |
| No | 12304 (82.1%) | 2461 (84.0%) | 3866 (83.8%) | 12315 (60.4%) | 1594 (58.8%) | 2142 (63.3%) |
| Planning to | 228 (1.5%) | 49 (1.7%) | 89 (1.9%) | 313 (1.5%) | 37 (1.4%) | 61 (1.8%) |
| Yes | 232 (1.5%) | 56 (1.9%) | 84 (1.8%) | 400 (2.0%) | 68 (2.5%) | 82 (2.4%) |
| Allready approved | 1236 (8.2%) | 197 (6.7%) | 324 (7.0%) | 2764 (13.6%) | 440 (16.2%) | 511 (15.1%) |
| Previously operated, percent |  |  |  |  |  |  |
| *Missing, n (%)* | *79 (0.5%)* | *23 (0.8%)* | *25 (0.5%)* | *133 (0.7%)* | *23 (0.8%)* | *31 (0.9%)* |
| Yes | 3124 (20.9%) | 667 (22.8%) | 1148 (24.9%) | 5022 (24.6%) | 742 (27.4%) | 945 (27.9%) |
| No | 11780 (78.6%) | 2240 (76.5%) | 3439 (74.6%) | 15222 (74.7%) | 1947 (71.8%) | 2408 (71.2%) |
| Number of previous operations, mean (SD) | 0.25 (0.59) | 0.28 (0.62) | 0.31 (0.65) | 0.32 (0.68) | 0.35 (0.70) | 0.37 (0.73) |
| *Missing, n (%)* | *369 (2.5%)* | *95 (3.2%)* | *129 (2.8%)* | *600 (2.9%)* | *81 (3.0%)* | *111 (3.3%)* |
| Has other relevant diseases, percent |  |  |  |  |  |  |
| *Missing, n (%)* | *1099 (7.3%)* | *242 (8.3%)* | *325 (7.0%)* | *1060 (5.2%)* | *116 (4.3%)* | *198 (5.9%)* |
| Yes | 4958 (33.1%) | 874 (29.8%) | 1303 (28.3%) | 13241 (65.0%) | 1756 (64.7%) | 2040 (60.3%) |
| No | 8926 (59.6%) | 1814 (61.9%) | 2984 (64.7%) | 6076 (29.8%) | 840 (31.0%) | 1146 (33.9%) |
| Degree of paresis, percent |  |  |  |  |  |  |
| *Missing, n (%)* | *0 (0.0%)* | *0 (0.0%)* | *0 (0.0%)* | *0 (0.0%)* | *0 (0.0%)* | *0 (0.0%)* |
| Total paralysis (0) | 47 (0.3%) | 9 (0.3%) | 13 (0.3%) | 72 (0.4%) | 9 (0.3%) | 14 (0.4%) |
| Palpable or visible contraction (1) | 41 (0.3%) | 2 (0.1%) | 6 (0.1%) | 40 (0.2%) | 6 (0.2%) | 9 (0.3%) |
| Active movement, gravity eliminated (2) | 88 (0.6%) | 9 (0.3%) | 14 (0.3%) | 62 (0.3%) | 9 (0.3%) | 10 (0.3%) |
| Active movement, against gravity (3) | 422 (2.8%) | 70 (2.4%) | 96 (2.1%) | 198 (1.0%) | 30 (1.1%) | 41 (1.2%) |
| Active movement, against some resistance (4) | 1321 (8.8%) | 270 (9.2%) | 373 (8.1%) | 795 (3.9%) | 111 (4.1%) | 133 (3.9%) |
| Active movement, against full resistance (5) | 324 (2.2%) | 57 (1.9%) | 101 (2.2%) | 335 (1.6%) | 45 (1.7%) | 44 (1.3%) |
| No symptoms (6) | 12740 (85.0%) | 2513 (85.8%) | 4009 (86.9%) | 18875 (92.6%) | 2502 (92.3%) | 3133 (92.6%) |
| ASA score, percent |  |  |  |  |  |  |
| *Missing, n (%)* | *186 (1.2%)* | *40 (1.4%)* | *56 (1.2%)* | *289 (1.4%)* | *38 (1.4%)* | *53 (1.6%)* |
| Normal health patient (1) | 6785 (45.3%) | 1405 (48.0%) | 2243 (48.6%) | 2776 (13.6%) | 418 (15.4%) | 581 (17.2%) |
| Patient with mild systemic disease (2) | 6946 (46.4%) | 1310 (44.7%) | 2109 (45.7%) | 12772 (62.7%) | 1604 (59.1%) | 2053 (60.7%) |
| Patient with severe systemic disease (3) | 1054 (7.0%) | 172 (5.9%) | 199 (4.3%) | 4476 (22.0%) | 643 (23.7%) | 688 (20.3%) |
| Patient with severe systemic disease that is a constant threat to life (4) | 11 (0.1%) | 3 (0.1%) | 5 (0.1%) | 60 (0.3%) | 9 (0.3%) | 9 (0.3%) |
| Moribund patient not expected to survive without the operation (5) | 1 (0.0%) | 0 (0.0%) | 0 (0.0%) | 4 (0.0%) | 0 (0.0%) | 0 (0.0%) |
