# Supplementary material for: Prediction of Oswestry Disability Index and Numeric Rating Scale scores after lumbar spine surgery: machine learning model development and fairness assessment
Source: BMJ Open. 2026 May 13;16(5):e108947. doi: 10.1136/bmjopen-2025-108947 (PMC13182469; doi:10.1136/bmjopen-2025-108947)
Supplement: online supplemental file 4 [file bmjopen-16-5-s004.docx]

# Table S4 Baseline characteristics of cases used for training NRS leg pain models

| Variables at baseline | LDH | | | LSS | | |
| --- | --- | --- | --- | --- | --- | --- |
|  | Included (n=17897) | | Excluded due to missing follow-up NRS (n=4628) | Included (n=22763) | | Excluded due to missing follow-up NRS (n=3403) |
|  | Cases with available 12-month NRS leg pain (n=14955) | Cases with available 3-month, but not 12-month NRS leg pain (n=2942) |  | Cases with available 12-month NRS leg pain (n=20007) | Cases with available 3-month, but not 12-month NRS leg pain (n=2756) |  |
| Sex, percent female |  |  |  |  |  |  |
| *Missing, n (%)* | *0 (0.0%)* | *0 (0.0%)* | *0 (0.0%)* | *0 (0.0%)* | *0 (0.0%)* | *0 (0.0%)* |
| Female | 6398 (42.8%) | 1176 (40.0%) | 1626 (35.1%) | 10132 (50.6%) | 1411 (51.2%) | 1678 (49.3%) |
| Male | 8557 (57.2%) | 1766 (60.0%) | 3002 (64.9%) | 9875 (49.4%) | 1345 (48.8%) | 1725 (50.7%) |
| Age, mean (SD) | 48.81 (14.28) | 44.43 (13.54) | 41.38 (12.57) | 65.81 (11.30) | 63.55 (13.07) | 60.74 (13.70) |
| *Missing, n (%)* | *39 (0.3%)* | *16 (0.5%)* | *22 (0.5%)* | *29 (0.1%)* | *2 (0.1%)* | *10 (0.3%)* |
| Smoking status, percent |  |  |  |  |  |  |
| *Missing, n (%)* | *127 (0.8%)* | *24 (0.8%)* | *51 (1.1%)* | *186 (0.9%)* | *25 (0.9%)* | *41 (1.2%)* |
| Yes | 3176 (21.2%) | 830 (28.2%) | 1383 (29.9%) | 3428 (17.1%) | 624 (22.6%) | 923 (27.1%) |
| No | 11652 (77.9%) | 2088 (71.0%) | 3194 (69.0%) | 16393 (81.9%) | 2107 (76.5%) | 2439 (71.7%) |
| BMI, mean (SD) | 26.90 (4.34) | 27.09 (4.49) | 27.22 (4.52) | 27.70 (4.40) | 27.73 (4.59) | 27.98 (4.70) |
| *Missing, n (%)* | *843 (5.6%)* | *163 (5.5%)* | *246 (5.3%)* | *806 (4.0%)* | *121 (4.4%)* | *157 (4.6%)* |
| Symptom duration (back or hip pain), percent |  |  |  |  |  |  |
| *Missing, n (%)* | *462 (3.1%)* | *89 (3.0%)* | *164 (3.5%)* | *799 (4.0%)* | *108 (3.9%)* | *137 (4.0%)* |
| No symptoms | 0 (0.0%) | 0 (0.0%) | 0 (0.0%) | 0 (0.0%) | 0 (0.0%) | 0 (0.0%) |
| 0 to 3 months | 403 (2.7%) | 55 (1.9%) | 95 (2.1%) | 408 (2.0%) | 51 (1.9%) | 51 (1.5%) |
| 3 to 12 months | 1563 (10.5%) | 279 (9.5%) | 448 (9.7%) | 421 (2.1%) | 56 (2.0%) | 73 (2.1%) |
| 12 to 24 months | 6868 (45.9%) | 1361 (46.3%) | 2099 (45.4%) | 4357 (21.8%) | 572 (20.8%) | 699 (20.5%) |
| More than 24 months | 2341 (15.7%) | 488 (16.6%) | 756 (16.3%) | 4172 (20.9%) | 559 (20.3%) | 733 (21.5%) |
| Symptom duration (leg pain), percent |  |  |  |  |  |  |
| *Missing, n (%)* | *557 (3.7%)* | *115 (3.9%)* | *204 (4.4%)* | *1035 (5.2%)* | *166 (6.0%)* | *195 (5.7%)* |
| No symptoms | 0 (0.0%) | 0 (0.0%) | 0 (0.0%) | 0 (0.0%) | 0 (0.0%) | 0 (0.0%) |
| 0 to 3 months | 199 (1.3%) | 34 (1.2%) | 69 (1.5%) | 625 (3.1%) | 71 (2.6%) | 87 (2.6%) |
| 3 to 12 months | 2382 (15.9%) | 423 (14.4%) | 659 (14.2%) | 729 (3.6%) | 107 (3.9%) | 132 (3.9%) |
| 12 to 24 months | 7860 (52.6%) | 1556 (52.9%) | 2384 (51.5%) | 5897 (29.5%) | 759 (27.5%) | 922 (27.1%) |
| More than 24 months | 2081 (13.9%) | 443 (15.1%) | 680 (14.7%) | 4808 (24.0%) | 672 (24.4%) | 808 (23.7%) |
| Use of painkillers, percent |  |  |  |  |  |  |
| *Missing, n (%)* | *53 (0.4%)* | *9 (0.3%)* | *25 (0.5%)* | *118 (0.6%)* | *10 (0.4%)* | *24 (0.7%)* |
| No | 2308 (15.4%) | 429 (14.6%) | 708 (15.3%) | 3875 (19.4%) | 471 (17.1%) | 607 (17.8%) |
| Yes | 12594 (84.2%) | 2504 (85.1%) | 3895 (84.2%) | 16014 (80.0%) | 2275 (82.5%) | 2772 (81.5%) |
| NRS back pain, mean (SD) | 6.11 (2.36) | 6.23 (2.33) | 6.19 (2.29) | 6.51 (2.18) | 6.67 (2.16) | 6.65 (2.18) |
| *Missing, n (%)* | *149 (1.0%)* | *22 (0.7%)* | *43 (0.9%)* | *368 (1.8%)* | *58 (2.1%)* | *51 (1.5%)* |
| NRS leg pain, mean (SD) | 6.73 (2.15) | 6.73 (2.15) | 6.68 (2.13) | 6.57 (2.22) | 6.73 (2.22) | 6.60 (2.27) |
| *Missing, n (%)* | *0 (0.0%)* | *0 (0.0%)* | *0 (0.0%)* | *0 (0.0%)* | *0 (0.0%)* | *0 (0.0%)* |
| ODI, mean (SD) | 42.82 (16.72) | 42.86 (17.06) | 42.67 (16.93) | 38.64 (14.88) | 40.90 (15.09) | 41.58 (15.60) |
| *Missing, n (%)* | *29 (0.2%)* | *4 (0.1%)* | *18 (0.4%)* | *124 (0.6%)* | *30 (1.1%)* | *29 (0.9%)* |
| EQ5D index, mean (SD) | 0.36 (0.32) | 0.34 (0.32) | 0.35 (0.32) | 0.43 (0.29) | 0.39 (0.30) | 0.37 (0.30) |
| *Missing, n (%)* | *449 (3.0%)* | *103 (3.5%)* | *173 (3.7%)* | *956 (4.8%)* | *156 (5.7%)* | *208 (6.1%)* |
| EQ5D anxiety score, percent |  |  |  |  |  |  |
| *Missing, n (%)* | *197 (1.3%)* | *37 (1.3%)* | *88 (1.9%)* | *437 (2.2%)* | *70 (2.5%)* | *98 (2.9%)* |
| Not anxious or depressed (level 1) | 8367 (55.9%) | 1532 (52.1%) | 2420 (52.3%) | 11799 (59.0%) | 1460 (53.0%) | 1667 (49.0%) |
| Slightly anxious or depressed (level 2) | 1448 (9.7%) | 261 (8.9%) | 417 (9.0%) | 2256 (11.3%) | 307 (11.1%) | 326 (9.6%) |
| Moderately anxious or depressed (level 3) | 4399 (29.4%) | 968 (32.9%) | 1473 (31.8%) | 4913 (24.6%) | 778 (28.2%) | 1123 (33.0%) |
| Severely anxious or depressed (level 4) | 169 (1.1%) | 43 (1.5%) | 83 (1.8%) | 284 (1.4%) | 55 (2.0%) | 67 (2.0%) |
| Extremely anxious or depressed (level 5) | 375 (2.5%) | 101 (3.4%) | 147 (3.2%) | 318 (1.6%) | 86 (3.1%) | 122 (3.6%) |
| Civil status, percent |  |  |  |  |  |  |
| *Missing, n (%)* | *134 (0.9%)* | *21 (0.7%)* | *52 (1.1%)* | *175 (0.9%)* | *28 (1.0%)* | *35 (1.0%)* |
| Living alone | 3435 (23.0%) | 799 (27.2%) | 1395 (30.1%) | 5008 (25.0%) | 796 (28.9%) | 1107 (32.5%) |
| Cohabiting | 11386 (76.1%) | 2122 (72.1%) | 3181 (68.7%) | 14824 (74.1%) | 1932 (70.1%) | 2261 (66.4%) |
| Work status, percent |  |  |  |  |  |  |
| *Missing, n (%)* | *481 (3.2%)* | *75 (2.5%)* | *185 (4.0%)* | *937 (4.7%)* | *105 (3.8%)* | *199 (5.8%)* |
| Working or student | 4080 (27.3%) | 777 (26.4%) | 1312 (28.3%) | 3342 (16.7%) | 494 (17.9%) | 607 (17.8%) |
| Retired | 1758 (11.8%) | 201 (6.8%) | 173 (3.7%) | 9491 (47.4%) | 1121 (40.7%) | 1106 (32.5%) |
| Sick leave | 6607 (44.2%) | 1476 (50.2%) | 2235 (48.3%) | 2923 (14.6%) | 464 (16.8%) | 741 (21.8%) |
| Unemployed / Work settlement allowance / Disability pension | 2029 (13.6%) | 413 (14.0%) | 723 (15.6%) | 3314 (16.6%) | 572 (20.8%) | 750 (22.0%) |
| Education, percent |  |  |  |  |  |  |
| *Missing, n (%)* | *167 (1.1%)* | *31 (1.1%)* | *76 (1.6%)* | *595 (3.0%)* | *84 (3.0%)* | *125 (3.7%)* |
| Elementary school, 7-10 years | 2015 (13.5%) | 412 (14.0%) | 653 (14.1%) | 4196 (21.0%) | 633 (23.0%) | 782 (23.0%) |
| High school | 6765 (45.2%) | 1422 (48.3%) | 2314 (50.0%) | 8420 (42.1%) | 1160 (42.1%) | 1513 (44.5%) |
| University <4 years | 3136 (21.0%) | 583 (19.8%) | 892 (19.3%) | 3671 (18.3%) | 474 (17.2%) | 544 (16.0%) |
| University >5 years | 2872 (19.2%) | 494 (16.8%) | 693 (15.0%) | 3125 (15.6%) | 405 (14.7%) | 439 (12.9%) |
| Applied for disability benefits, percent |  |  |  |  |  |  |
| *Missing, n (%)* | *971 (6.5%)* | *167 (5.7%)* | *245 (5.3%)* | *4439 (22.2%)* | *576 (20.9%)* | *586 (17.2%)* |
| No | 12308 (82.3%) | 2474 (84.1%) | 3888 (84.0%) | 12149 (60.7%) | 1635 (59.3%) | 2154 (63.3%) |
| Planning to | 224 (1.5%) | 50 (1.7%) | 90 (1.9%) | 312 (1.6%) | 37 (1.3%) | 61 (1.8%) |
| Yes | 230 (1.5%) | 57 (1.9%) | 82 (1.8%) | 390 (1.9%) | 66 (2.4%) | 82 (2.4%) |
| Allready approved | 1222 (8.2%) | 194 (6.6%) | 323 (7.0%) | 2717 (13.6%) | 442 (16.0%) | 520 (15.3%) |
| Previously operated, percent |  |  |  |  |  |  |
| *Missing, n (%)* | *74 (0.5%)* | *23 (0.8%)* | *27 (0.6%)* | *131 (0.7%)* | *25 (0.9%)* | *30 (0.9%)* |
| Yes | 3118 (20.8%) | 668 (22.7%) | 1153 (24.9%) | 4937 (24.7%) | 753 (27.3%) | 943 (27.7%) |
| No | 11763 (78.7%) | 2251 (76.5%) | 3448 (74.5%) | 14939 (74.7%) | 1978 (71.8%) | 2430 (71.4%) |
| Number of previous operations, mean (SD) | 0.25 (0.59) | 0.28 (0.62) | 0.31 (0.65) | 0.32 (0.68) | 0.36 (0.70) | 0.37 (0.72) |
| *Missing, n (%)* | *363 (2.4%)* | *95 (3.2%)* | *132 (2.9%)* | *586 (2.9%)* | *82 (3.0%)* | *114 (3.3%)* |
| Has other relevant diseases, percent |  |  |  |  |  |  |
| *Missing, n (%)* | *1090 (7.3%)* | *244 (8.3%)* | *327 (7.1%)* | *1039 (5.2%)* | *125 (4.5%)* | *200 (5.9%)* |
| Yes | 4930 (33.0%) | 885 (30.1%) | 1302 (28.1%) | 12948 (64.7%) | 1769 (64.2%) | 2047 (60.2%) |
| No | 8935 (59.7%) | 1813 (61.6%) | 2999 (64.8%) | 6020 (30.1%) | 862 (31.3%) | 1156 (34.0%) |
| Degree of paresis, percent |  |  |  |  |  |  |
| *Missing, n (%)* | *0 (0.0%)* | *0 (0.0%)* | *0 (0.0%)* | *0 (0.0%)* | *0 (0.0%)* | *0 (0.0%)* |
| Total paralysis (0) | 45 (0.3%) | 8 (0.3%) | 13 (0.3%) | 69 (0.3%) | 8 (0.3%) | 12 (0.4%) |
| Palpable or visible contraction (1) | 41 (0.3%) | 2 (0.1%) | 6 (0.1%) | 38 (0.2%) | 6 (0.2%) | 8 (0.2%) |
| Active movement, gravity eliminated (2) | 88 (0.6%) | 11 (0.4%) | 14 (0.3%) | 61 (0.3%) | 9 (0.3%) | 10 (0.3%) |
| Active movement, against gravity (3) | 421 (2.8%) | 71 (2.4%) | 97 (2.1%) | 197 (1.0%) | 33 (1.2%) | 37 (1.1%) |
| Active movement, against some resistance (4) | 1325 (8.9%) | 273 (9.3%) | 378 (8.2%) | 791 (4.0%) | 112 (4.1%) | 134 (3.9%) |
| Active movement, against full resistance (5) | 323 (2.2%) | 56 (1.9%) | 98 (2.1%) | 328 (1.6%) | 47 (1.7%) | 47 (1.4%) |
| No symptoms (6) | 12712 (85.0%) | 2521 (85.7%) | 4022 (86.9%) | 18523 (92.6%) | 2541 (92.2%) | 3155 (92.7%) |
| ASA score, percent |  |  |  |  |  |  |
| *Missing, n (%)* | *183 (1.2%)* | *40 (1.4%)* | *56 (1.2%)* | *287 (1.4%)* | *39 (1.4%)* | *53 (1.6%)* |
| Normal health patient (1) | 6789 (45.4%) | 1402 (47.7%) | 2248 (48.6%) | 2750 (13.7%) | 432 (15.7%) | 589 (17.3%) |
| Patient with mild systemic disease (2) | 6940 (46.4%) | 1332 (45.3%) | 2120 (45.8%) | 12538 (62.7%) | 1633 (59.3%) | 2071 (60.9%) |
| Patient with severe systemic disease (3) | 1032 (6.9%) | 165 (5.6%) | 200 (4.3%) | 4368 (21.8%) | 643 (23.3%) | 682 (20.0%) |
| Patient with severe systemic disease that is a constant threat to life (4) | 10 (0.1%) | 3 (0.1%) | 4 (0.1%) | 60 (0.3%) | 9 (0.3%) | 8 (0.2%) |
| Moribund patient not expected to survive without the operation (5) | 1 (0.0%) | 0 (0.0%) | 0 (0.0%) | 4 (0.0%) | 0 (0.0%) | 0 (0.0%) |
