# Supplementary material for: Prediction of Oswestry Disability Index and Numeric Rating Scale scores after lumbar spine surgery: machine learning model development and fairness assessment
Source: BMJ Open. 2026 May 13;16(5):e108947. doi: 10.1136/bmjopen-2025-108947 (PMC13182469; doi:10.1136/bmjopen-2025-108947)
Supplement: online supplemental file 5 [file bmjopen-16-5-s005.docx]

# Table S5 – Hyperparameters selected for each model

| Model | Learning rate | Interaction depth | Total estimators |
| --- | --- | --- | --- |
| LDH | | | |
| ODI | 0.1 | 4 | 100 |
| NRS leg pain | 0.1 | 3 | 100 |
| NRS back pain | 0.1 | 2 | 200 |
| LSS | | | |
| ODI | 0.1 | 3 | 200 |
| NRS leg pain | 0.1 | 1 | 500 |
| NRS back pain | 0.01 | 4 | 500 |
