# Supplementary material for: Prediction of Oswestry Disability Index and Numeric Rating Scale scores after lumbar spine surgery: machine learning model development and fairness assessment
Source: BMJ Open. 2026 May 13;16(5):e108947. doi: 10.1136/bmjopen-2025-108947 (PMC13182469; doi:10.1136/bmjopen-2025-108947)
Supplement: online supplemental file 6 [file bmjopen-16-5-s006.docx]

# Table S6 Results of different models where cases with available 3 month follow-up but missing 12-month follow-up were imputed with last carried forward

| Model | MAE  [estimation, lower confidence interval, upper confidence interval] | MSE | R2 | auroc_CI | PR_ROC_CI | sensitivity | specificity |
| --- | --- | --- | --- | --- | --- | --- | --- |
| LDH_OSW_training | [10.908, 10.773, 11.041] | [197.097, 191.837, 202.271] | [0.309, 0.296, 0.322] | [0.843, 0.837, 0.848] | [0.863, 0.856, 0.87] | 0,753967 | 0,764507 |
| LDH_OSW_testing | [11.32, 11.001, 11.628] | [211.725, 199.486, 223.75] | [0.265, 0.236, 0.294] | [0.824, 0.811, 0.839] | [0.852, 0.834, 0.869] | 0,725468 | 0,764617 |
| LDH_LEG_training | [1.927, 1.907, 1.949] | [5.554, 5.443, 5.674] | [0.21, 0.198, 0.22] | [0.817, 0.811, 0.824] | [0.751, 0.74, 0.762] | 0,551138 | 0,847706 |
| LDH_LEG_testing | [1.945, 1.9, 1.994] | [5.6, 5.351, 5.86] | [0.21, 0.186, 0.231] | [0.82, 0.804, 0.834] | [0.755, 0.732, 0.781] | 0,551128 | 0,845831 |
| LDH_BACK_training | [2.002, 1.979, 2.026] | [6.064, 5.931, 6.197] | [0.173, 0.162, 0.183] | [0.771, 0.762, 0.78] | [0.912, 0.907, 0.918] | 0,915802 | 0,390354 |
| LDH_BACK_testing | [2.093, 2.042, 2.15] | [6.647, 6.319, 6.994] | [0.166, 0.141, 0.188] | [0.743, 0.723, 0.763] | [0.891, 0.877, 0.904] | 0,907618 | 0,355021 |
| LSS_OSW_training | [11.908, 11.783, 12.04] | [221.396, 216.771, 226.05] | [0.333, 0.322, 0.345] | [0.722, 0.715, 0.729] | [0.724, 0.715, 0.734] | 0,649602 | 0,662304 |
| LSS_OSW_testing | [12.046, 11.76, 12.319] | [227.75, 217.802, 237.722] | [0.31, 0.284, 0.337] | [0.716, 0.701, 0.731] | [0.725, 0.706, 0.744] | 0,663265 | 0,654773 |
| LSS_LEG_training | [2.109, 2.088, 2.129] | [6.34, 6.24, 6.449] | [0.192, 0.182, 0.202] | [0.748, 0.741, 0.755] | [0.604, 0.592, 0.616] | 0,31992 | 0,897376 |
| LSS_LEG_testing | [2.125, 2.081, 2.164] | [6.369, 6.144, 6.585] | [0.188, 0.167, 0.209] | [0.735, 0.718, 0.749] | [0.604, 0.576, 0.633] | 0,325988 | 0,892223 |
| LSS_BACK_training | [2.326, 2.304, 2.346] | [7.486, 7.368, 7.592] | [0.145, 0.136, 0.154] | [0.713, 0.705, 0.721] | [0.809, 0.801, 0.817] | 0,845163 | 0,425732 |
| LSS_BACK_testing | [2.329, 2.283, 2.376] | [7.543, 7.303, 7.804] | [0.115, 0.095, 0.135] | [0.709, 0.691, 0.727] | [0.811, 0.793, 0.829] | 0,847655 | 0,433059 |
