# Supplementary material for: Prediction of Oswestry Disability Index and Numeric Rating Scale scores after lumbar spine surgery: machine learning model development and fairness assessment
Source: BMJ Open. 2026 May 13;16(5):e108947. doi: 10.1136/bmjopen-2025-108947 (PMC13182469; doi:10.1136/bmjopen-2025-108947)
Supplement: online supplemental file 7 [file bmjopen-16-5-s007.docx]

# Table S7 Results of different models without last-carried-forward imputation

| Model | MAE  [estimation, lower confidence interval, upper confidence interval] | MSE | R2 | auroc_CI | PR_ROC_CI | sensitivity | specificity |
| --- | --- | --- | --- | --- | --- | --- | --- |
| LDH_OSW_training | [10.665, 10.515, 10.823] | [189.838, 183.967, 196.305] | [0.32, 0.305, 0.335] | [0.847, 0.84, 0.855] | [0.874, 0.866, 0.881] | 0,771038 | 0,761284 |
| LDH_OSW_testing | [11.266, 10.952, 11.58] | [212.842, 200.354, 225.756] | [0.261, 0.23, 0.291] | [0.823, 0.808, 0.836] | [0.85, 0.832, 0.867] | 0,744186 | 0,749431 |
| LDH_LEG_training | [1.931, 1.906, 1.954] | [5.574, 5.448, 5.702] | [0.214, 0.201, 0.226] | [0.82, 0.813, 0.828] | [0.756, 0.744, 0.769] | 0,564127 | 0,8462 |
| LDH_LEG_testing | [1.933, 1.884, 1.981] | [5.576, 5.318, 5.845] | [0.213, 0.189, 0.238] | [0.82, 0.806, 0.834] | [0.757, 0.731, 0.782] | 0,562406 | 0,836833 |
| LDH_BACK_training | [1.968, 1.942, 1.995] | [5.901, 5.753, 6.053] | [0.19, 0.177, 0.202] | [0.781, 0.771, 0.792] | [0.919, 0.913, 0.925] | 0,917176 | 0,406309 |
| LDH_BACK_testing | [2.076, 2.024, 2.129] | [6.62, 6.294, 6.971] | [0.169, 0.147, 0.192] | [0.746, 0.726, 0.765] | [0.892, 0.878, 0.906] | 0,909807 | 0,353607 |
| LSS_OSW_training | [11.771, 11.634, 11.903] | [215.642, 210.943, 220.298] | [0.344, 0.333, 0.356] | [0.727, 0.719, 0.734] | [0.736, 0.727, 0.746] | 0,663309 | 0,654706 |
| LSS_OSW_testing | [12.027, 11.751, 12.319] | [226.882, 217.773, 236.756] | [0.313, 0.288, 0.339] | [0.716, 0.701, 0.731] | [0.726, 0.706, 0.745] | 0,667258 | 0,639826 |
| LSS_LEG_training | [2.103, 2.08, 2.124] | [6.319, 6.208, 6.431] | [0.2, 0.191, 0.211] | [0.751, 0.744, 0.758] | [0.613, 0.6, 0.627] | 0,337017 | 0,893837 |
| LSS_LEG_testing | [2.12, 2.075, 2.162] | [6.362, 6.137, 6.581] | [0.189, 0.169, 0.209] | [0.735, 0.721, 0.752] | [0.607, 0.581, 0.635] | 0,338302 | 0,891828 |
| LSS_BACK_training | [2.276, 2.254, 2.297] | [7.214, 7.092, 7.336] | [0.174, 0.164, 0.183] | [0.725, 0.718, 0.734] | [0.821, 0.814, 0.829] | 0,846695 | 0,441483 |
| LSS_BACK_testing | [2.322, 2.277, 2.367] | [7.563, 7.297, 7.832] | [0.113, 0.09, 0.135] | [0.709, 0.691, 0.725] | [0.811, 0.794, 0.828] | 0,842026 | 0,428571 |
