# Supplementary material for: Prediction of Oswestry Disability Index and Numeric Rating Scale scores after lumbar spine surgery: machine learning model development and fairness assessment
Source: BMJ Open. 2026 May 13;16(5):e108947. doi: 10.1136/bmjopen-2025-108947 (PMC13182469; doi:10.1136/bmjopen-2025-108947)
Supplement: online supplemental file 8 [file bmjopen-16-5-s008.docx]

# Figure S1 – Normalized L1 distance for patients in the test set, per model

*Figure S1: These violin plots show the distribution of normalized L1 distance to the nearest 50 patients in the training set, for patients in the test set. Mean distance for the entire test set is shown as a bold line within each violin.*
