# Supplementary material for: Prediction of Oswestry Disability Index and Numeric Rating Scale scores after lumbar spine surgery: machine learning model development and fairness assessment
Source: BMJ Open. 2026 May 13;16(5):e108947. doi: 10.1136/bmjopen-2025-108947 (PMC13182469; doi:10.1136/bmjopen-2025-108947)
Supplement: Supplementary file 9 [file bmjopen-2025-108947.reviewer_comments.pdf]

PEER REVIEW HISTORY

BMJ Open publishes all reviews undertaken for accepted manuscripts. Reviewers are asked to complete a checklist review form and are provided with free text boxes to elaborate on their assessment. These free text comments are reproduced below.

ARTICLE DETAILS

Title (Provisional)

Prediction of Oswestry disability index and numeric rating scale scores after lumbar spine surgery: machine learning model development and fairness assessment

Authors

Joakimsen, Henrik Lykke; Lund, Jørgen Aarmo; Burman, Joel; Woldaregay, Ashenafi Zebene; Berg , Bjørnar; Solberg, Tore K; Ingebrigtsen, Tor; Mikalsen, Karl Øyvind

---

VERSION 1 - REVIEW

**Reviewer**	**1**
**Name**	**Kelson, Zoe**
**Affiliation**	**University of Exeter, Mathematics**
**Date**	**22-Sep-2025**
**COI**	**None**

This study aims to develop and test models for prediction of ordinal outcome scale scores and retrieval of similar patients' outcomes, and to evaluate the models' fairness.

Reviewer comments:

"This study aligns with the recommendation of Transparent reporting of a multivariable prediction model for individual prognosis or diagnosis statement for reporting clinical prediction models that use machine learning (TRIPOD+AI)."

Thanks for providing a copy of the TRIPOD+AI checklist.

"All cases recorded with an elective operation for lumbar disc herniation (LDH, n=17,880) or spinal stenosis (LSS, n=23,697) in the Norwegian registry for spine surgery from 2007 through 2023" [Abstract]

and

"We used data recorded in NORspine from January 1st, 2007, to June 23rd, 2023. NORspine comprises a cohort of 69,664 cases registered from all 41 public and private hospitals in Norway providing spine surgery. It had a capture rate of 82%, a 3-month follow-up rate of 80% and 12-month follow-up of 72% in 2023"

Can the authors please further comment on whether the included cohorts can be considered to be representative?

Can the authors please consider the impact of COVID-19 on the study?

"Reoperation within 90 days is recorded as treatment of a complication, while new spine surgery after 90 days is considered as a new case and initiates new 3- and 12-month follow-ups"

Can the authors explore whether a sensitivity analysis on this definition might be of interest?

"All outcomes were patient-reported 12 months after the operation"

Can the authors please comment on the potential for self reporting bias?

"We selected 22 predictors recorded preoperatively by patients and clinicians based on Shapley additive explanation values." [Abstract]

and

"All available presurgical features in NORspine (listed in Table S1 in the supplementary file) were assessed as potential predictors. Initially, assessment was conducted by training models using all presurgical features. We then measured each feature's contribution to the final predictive model by their Shapley additive explanations (SHAP) values, with the method proposed by Lundberg and Lee. [27] From this, a subset of candidate features was selected and reviewed with experienced spine surgeons (TS and TI) and a lawyer (MH) regarding clinical relevance and legality in potential subsequent decision support product development."

The authors do well to demonstrate that included features are statistically important and clinically meaningful.

"For sample size calculations, we assumed our prediction models to reach an adjusted R2 of 0.20, intercept of 24.0, and standard error of 0.4, using a maximum of 50 parameters. This corresponded to a requirement of minimum 1,924 cases for training"

The authors have suitably communicated the basis for the sample size calculation.

Can the associated assessment of study power please be reported here too?

"Patients with missing outcome data were excluded listwise, i.e., missing pre- (n=877) or postoperative (n=12,486) ODI scores, back pain (pre- n=4,234, postoperative n=14,616), or leg pain (pre- n=3,667, postoperative n=15,204) scores."

Can the authors please comment on whether missing outcome data can be considered to be missing at random?

"Missing values in NORspine were considered missing at random. [31] There are some instructions and definitions in the registry that are useful to be aware of when assessing missing data. Degree of paresis is assumed to be normal if missing. If whether a patient has

applied for disability benefits is missing, it is assumed they have not applied. Otherwise, missing data were imputed using a K nearest neighbour (KNN) imputer. [32] After initial experiments, values of K in the range 3 to 200 were found satisfactory and further tested to find an optimal threshold. Optimal K was chosen by balancing performance and complexity based on repeated experiments and expert assessment. We weighted towards lower K, i.e., where the curve of performance flattened. Finally, we compared models trained on imputed data to models trained without imputation, as well as to models trained on data imputed with mean value of each feature. "

The authors have satisfactorily handled missing data, and conducted a number of valuable sensitivity analyses on the approach taken.

"For cases with available 3-month but missing 12-month scores (ODI n=11,068, back pain n= 8,027, leg pain n=8,201), imputation was done by last observation carried forward. These cases were not used in the test set."

Can the authors please explore a sensitivity analysis on the treatment of 'available 3-month but missing 12-month scores'?

"Data were split in 80/20% training/test samples for LDH and LSS...

Six machine learning methods (extreme gradient boosting (XGBoost), Gaussian process regression, gradient boosting regression, artificial neural network, and linear regression) were trained for both conditions using 5-fold cross validation." [Abstract]

and

"To validate model structures, each dataset was divided into an 80/20% training/test split. All variables in both datasets were then normalized...

5-fold cross validation was used on the training data to train six different statistical and AI-enabled methods: linear regression, gradient boosting regression, extreme gradient boosting (XGBoost), Gaussian process regression and artificial neural networks...

For sensitivity analyses, the regression models were tested in a classification setting by reinterpreting the predicted ODI scores as estimates of the probability of success. "

Appropriate modelling methods have been applied, which are clearly described by the authors.

"We report the magnitude and distribution of errors as mean absolute error (MAE) with 95% confidence intervals (CI), and explanatory power as the coefficient of determination (R 2). Fairness and calibration were assessed with violin and calibration plots. "

Suitable performance metrics have been assessed.

"The models only predict outcomes of surgical treatment, and does not compare with outcomes of non-surgical treatment" [Strengths and Limitations of this Study]

and

"8.2 Limitations"

The authors provide a discussion on the study limitations.

**Reviewer**	**2**
**Name**	**Canizares, Mayilee**
**Affiliation**	**UHN, Arthritis Program**
**Date**	**26-Jan-2026**
**COI**	**None**

The manuscript "Prediction of Oswestry disability index and numeric rating scale scores after lumbar spine surgery: machine learning model development and fairness assessment" used machine learning techniques to develop prediction models for ODI and pain scores for patients undergoing elective surgery for LDH or LSS. The manuscript is well-organized and detailed. The methods seem generally appropriate but there are areas requiring clarifications.

- Throughout the manuscript the authors referred to the ODI (0-100) as ordinal scale and the pain scores (0-10) as numeric. From what I understand from the result section, both were modeled as continuous variables (i.e., assumed normal or other Gaussian distribution in the regression models) therefore they should be described as continuous variables.
- Could the authors expand on the statement that models performed well as errors were below the minimal clinical important change? MCIC are meant to be used at the individual level as applying the thresholds to the average of an entire group potentially mask individual level change.
- In the Introduction (lines 15-24) the authors make the case for the drawbacks in using binary outcomes based on a response classifier, but in their methodology one measure to evaluate the performance of their continuous models was MCIC thresholds. Please elaborate on the rationale behind this.
- Page 6, last paragraph: I think it is important to distinguish from patients who were not yet eligible for follow-up to those who did not respond or were lost to follow up. Furthermore, I suggest including a table with basic comparisons of baseline characteristics between those included and excluded (eligible but with missing data or lost to follow-up). This is important for biases assessment.
- Page 9, line 16: The variables were standardized to mean=0 and std. dev=1 but this does not guarantee normal distribution. Since this is a linear transformation, the standardized variable will have the same distribution as the original variable. Please correct the statement.
- Was pre-surgery ODI or pain included as predictive variables in the models?
- Sensitivity analysis: Please clarify the meaning of probability of success. Were models fit with a binary outcome? Or the % deemed successful was determined by calculating the change between pre-surgery ODI and predicted 12 month ODI and then applying the MCIC threshold for the classification? From the 2nd sentence in this paragraph it seems that what was modeled was the change in ODI which is not a probability.
- Page 10, Please clarify "By reinterpreting the regression estimates as class probabilities, we found the receiver operating curve (ROC) by computing the true positive..."

- Page 11, “Class imbalance”: Please add in the sensitivity analysis section that binary models were built.
- Table 1 & 2, I think that the information in the last column for “use of painkillers” and “NRS back pain” are swapped.
- Table 3, Could the authors state which was the final model for each variable? Also, it would be helpful to see the variables retained in the final model
- Page 18, Sensitivity analysis: How many variables included in the final models (binary vs continuous) overlapped? Please include a table with this result in the supplement

---

VERSION 1 - AUTHOR RESPONSE

Response to Reviewer #1

1. **“All cases recorded with an elective operation for lumbar disc herniation (LDH, n=17,880) or spinal stenosis (LSS, n=23,697) in the Norwegian registry for spine surgery from 2007 through 2023" [Abstract] and "We used data recorded in NORspine from January 1st, 2007, to June 23rd, 2023. NORspine comprises a cohort of 69,664 cases registered from all 41 public and private hospitals in Norway providing spine surgery. It had a capture rate of 82%, a 3-month follow-up rate of 80% and 12-month follow-up of 72% in 2023"**
   Can the authors please further comment on whether the included cohorts can be considered to be representative?

   The cited text was our description of NORspine, which was our data-source, and not a description of the included cohorts. We have clarified by changing the title of subsection *4.1 Data* to *4.1 Data source*. We have also moved the previously introductory information in subsection 4.1 about the study period to subsection *4.2 Participants* and clarified that data about the study cohort was retrieved from NORspine (lines 184-186).

   NORspine calculates capture rates every second year by comparing patient administrative data (considered nearly 100 % complete because coding is prerequisite for reimbursement) and registry data. These comparisons also provide data for drop-out analyses at baseline, showing that the capture rate is lower for emergency than scheduled operations, and that mean age is lower for non-captured than captured cases. There is no sex-difference in capture rate. This information has been added and quantified based on a new reference in the first paragraph of section *4.1 Data source* (lines 153-159) in *Methods,* and accordingly in subsection *8.2 Limitations* in the *Discussion* (line 915 and lines 919-925). A capture rate above 80 % is generally considered sufficient to achieve a representative sample in clinical registries, but there is no substantial evidence for this recommendation in the literature (1).

   We thus consider the cohorts captured by NORspine to be representative for patients undergoing scheduled operations.

2. **Can the authors please consider the impact of COVID-19 on the study?**

   The COVID-19 pandemic in Norway started in February 2020 and prompted extraordinary infection control measures for two years, until February 2022. This did not influence the number of lumbar spine operations registered in NORspine much: n_{2019}=5319, n_{2020}=5204, n_{2021}=6005, and n_{2022}=6316. The increase from 2019 to 2022 can partly be attributed to an increase in the capture rate. Further considerations about the impact of the pandemic would be speculative, and we have therefore not done revisions in the manuscript.

3. **"Reoperation within 90 days is recorded as treatment of a complication, while new spine surgery after 90 days is considered as a new case and initiates new 3- and 12-month follow-ups".**
   Can the authors explore whether a sensitivity analysis on this definition might be of interest?

   The purpose of the prediction model is to inform shared decision making about the indication for surgical treatment. This requires use of predictors that are available at the time of pre-surgical assessment. Information about reoperations is an intermediate outcome, and not a predictor, and treatment of complications is an integral part of the surgical treatment process. A sensitivity analysis with exclusion of these patients from the model development would therefore not make sense.

   Sensitivity analyses exploring shorter cut-offs (e.g. 30 or 60 days) are not possible because new operations within 90 days do not initiate a new case in NORspine. In consequence, the register does not contain data for such analyses. The number undergoing early reoperation as a treatment of complications within 90 days was very low (139/18,377 (0,8 %) for LDH and 79/24,540 (0,3 % for LSS)) and we therefore consider this insignificant.

4. **"All outcomes were patient-reported 12 months after the operation"**
   Can the authors please comment on the potential for self reporting bias?

   In general, PROMs have some shortcomings but are considered to capture patient's individual experience of health and pain more accurately than physician reported outcomes. NORspine uses a set of validated and responsive patient reported outcome measures (PROM) recommended by the International consortium for health outcome measurement (ICHOM) to limit potential self-reporting bias (2). We have elaborated on this in paragraph three of section *4.1 Data source* (lines 169-172) in *Methods,* and accordingly in subsection *8.2 Limitations* in the *Discussion* (lines 933-934).

Some studies show systematic differences in the response to PROMs across sociodemographic subgroups, e.g. patients with higher age and lower education may respond towards ends of scales (3). It is therefore important to explore prediction models' performance across sociodemographic groups, as we have done in this work.

5. **"For sample size calculations, we assumed our prediction models to reach an adjusted R2 of 0.20, intercept of 24.0, and standard error of 0.4, using a maximum of 50 parameters. This corresponded to a requirement of minimum 1,924 cases for training"**
   The authors have suitably communicated the basis for the sample size calculation.
   Can the associated assessment of study power please be reported here too?

   We have added information about assessment of the study power in section *4.6 Sample size* (lines 238-243).

6. **"Patients with missing outcome data were excluded listwise, i.e., missing pre- (n=877) or postoperative (n=12,486) ODI scores, back pain (pre- n=4,234, postoperative n=14,616), or leg pain (pre- n=3,667, postoperative n=15,204) scores."**
   Can the authors please comment on whether missing outcome data can be considered to be missing at random?

   A previous study compared responders and non-responders at 12-month follow-up by contacting non-responder per telephone, and found that non-responders were younger and more likely to be smokers than responders, but there were no differences in outcomes (4). These findings suggest that loss to follow-up in NORspine is compatible with a missing-at-random assumption. We have added this information and the new reference in the first paragraph of section *4.1 Data source* (lines 153-159) in *Methods*.

   In the same study, the proportion of responders with missing data for the primary outcome (ODI) was very low (0.3 %), and the proportions for the secondary outcomes NRS leg- and back-pain were low (3.6 and 1.5 %, respectively) (4). We thus consider missing outcome data to be compatible with a missing at random assumption.

   The text in *4.2 Participants* is primarily about inclusion- and exclusion-criteria. We have therefore chosen to add supplemental information about missingness under section *4.7 Missing data* (lines 264-266).

7. **"For cases with available 3-month but missing 12-month scores (ODI n=11,068, back pain n= 8,027, leg pain n=8,201), imputation was done by last observation carried forward. These cases were not used in the test set."**
   Can the authors please explore a sensitivity analysis on the treatment of 'available 3-month but missing 12-month scores'?

   We have done the suggested analysis, and found minor non-significant differences compared to the main analysis.

   We have revised accordingly by adding the methodological description at the end of subsection *4.7 Missing data* in *Methods* (line 278-281). In *Results*, we report the sensitivity analysis in the second paragraph of subsection *7.4 Model performance* (lines 734-736) and in two new supplemental tables (tables S5 and S6).

Response to reviewer 2

1. **Throughout the manuscript the authors referred to the ODI (0-100) as ordinal scale and the pain scores (0-10) as numeric. From what I understand from the result section, both were modeled as continuous variables (i.e., assumed normal or other Gaussian distribution in the regression models) therefore they should be described as continuous variables.**

   Most clinical researchers consider the ODI and NRS as ordinal scales because they order categorical outcomes in numbered sequences. The reviewer is right, however, that we modelled the variables as continuous. This was explained in the third paragraph in subsection *4.8 Analytical methods* in *Methods* in the original manuscript and has been retained. We agree with the reviewer that when using this modelling, it is more correct to describe the outcomes as continuous and have revised accordingly throughout the manuscript.

2. **Could the authors expand on the statement that models performed well as errors were below the minimal clinical important change? MCIC are meant to be used at the individual level as applying the thresholds to the average of an entire group potentially mask individual level change.**

   We agree that the MCIC is not the best comparator when evaluating the clinical meaning of the MAEs for individual patients. We have therefore omitted the comparisons with MCICs and the four related references in the first paragraph of the *Discussion* and in the subsection *8.1 Prediction of continuous outcomes*, also in the D*iscussion*. Instead, we compare with published standard errors of measurements (SEM) for the instruments used to measure the outcomes (ODI and NRS) (two new references have been added), and discretionary assess the clinical meaning of the MAEs in this context (line 788-791). We have revised accordingly in the paragraph *Fairness* in the same subsection (line 875-876) and in the *Abstract* (line 78 and 81-

82).

3. **In the Introduction (lines 15-24) the authors make the case for the drawbacks in using binary outcomes based on a response classifier, but in their methodology one measure to evaluate the performance of their continuous models was MCIC thresholds. Please elaborate on the rationale behind this.**

   It is a misunderstanding that we used MCIC thresholds to evaluate the continuous models. We think the cause for this misunderstanding was that we mentioned our use of classification and calculation of AUC for sensitivity analysis in the description of the primary analysis in the first paragraph *Prediction of continuous outcomes* in subsection *4.8 Analytical methods*.

   To clarify, we have restructured by placing all information about sensitivity analyses in the paragraph *Sensitivity analyses* in the same subsection. In addition, the subsection *4.9 Class imbalance* was confusing, since considerations about possible correction methods for such imbalance were relevant only for the sensitivity analyses. We have therefore omitted this as a separate subsection and moved the text to the paragraph *Sensitivity analyses* in subsection *4.8 Analytical methods*. In addition, the new text in this paragraph (lines 350-364) has been revised in response to the same reviewer's comments no. 7, 8 and 9. Subsequent subsections have re-numbered accordingly.

4. **Page 6, last paragraph: I think it is important to distinguish from patients who were not yet eligible for follow-up to those who did not respond or were lost to follow up. Furthermore, I suggest including a table with basic comparisons of baseline characteristics between those included and excluded (eligible but with missing data or lost to follow-up). This is important for biases assessment.**

   Thank you for notifying about this important distinction. We agree that distinguishing between patients who were not yet eligible for follow-up and those who did not respond or were lost to follow up is very important. We designed the study to include only patients who were eligible for follow-up, and have clarified by adding more specific information about the timing of inclusion and follow-up in subsection *4.2 Participants* (lines 184-186).

   In response to this comment, we re-checked the study's patient flow and discovered that several hundred patients had been unintentionally excluded, mainly because of this error. In consequence, we had to retrain all the models. This caused small changes in all numbers throughout the *Abstract*, main text, all tables and *Figure 1 Flowchart of participants in modelling of ODI 12 months after surgery*. The performance estimates did not change significantly.

We agree that comparisons of baseline characteristics between included patients and patients excluded because they were lost to follow-up is important for assessment of bias, and have added the suggested table as a new *Table 1 Baseline characteristics of cases included in the ODI models development* in *Results*. In addition, we have added similar tables for cases included in the NRS back- and leg-pain models as *Supplementary tables S3 and S4.*

5. **Page 9, line 16: The variables were standardized to mean=0 and std. dev=1 but this does not guarantee normal distribution. Since this is a linear transformation, the standardized variable will have the same distribution as the original variable. Please correct the statement.**

   We agree and the statement has been corrected in the paragraph *Prediction of continuous outcomes* in subsection *4.8 Analytical methods* in *Methods* (line 286).

6. **Was pre-surgery ODI or pain included as predictive variables in the models?**

   Pre-surgery ODI and back- and leg-pain were included as predictive variables. This has been clarified by combining the earlier tables 1 and 2 into a new *Table 2 Predictors included in the ODI models* in *Results*. This table shows all the 22 predictors, and that they (randomly) were the same both for LDH and LSS. The text in subsection *7.1 Participants* in *Results* has been revised accordingly (line 469-475). To further clarify, we have added a reference to *Table 2* also in subsection *7.2 Model development* (line 481).

7. **Sensitivity analysis: Please clarify the meaning of probability of success. Were models fit with a binary outcome? Or the % deemed successful was determined by calculating the change between pre-surgery ODI and predicted 12 month ODI and then applying the MCIC threshold for the classification? From the 2nd sentence in this paragraph it seems that what was modelled was the change in ODI which is not a probability.**

   The models were not fit with a binary outcome. The regression models for prediction of continuous outcomes have few comparators in the literature. Therefore, in sensitivity analysis, we tested the regression models in a classification setting by applying previously published cut-offs for success versus non-success (and not MCIC thresholds) to the regression output (5). This has been clarified by the restructuring explained in our response to commentary no. 3 above and by the revision of the paragraph *Sensitivity analyses* in subsection *4.8 Analytical methods* in *Methods* (lines 350-3364). This includes adding a new reference to the method we used to calculate

the AUC. The corresponding paragraph in subsection *7.4 Model performance* in *Results* has been revised accordingly (line 746).

8. **Page 10, Please clarify "By reinterpreting the regression estimates as class probabilities, we found the receiver operating curve (ROC) by computing the true positive…"**

   This clarification is included in our response to commentary no. 7 above.

9. **Page 11, "Class imbalance": Please add in the sensitivity analysis section that binary models were built.**

   It is a misunderstanding that binary models were built. This too has been clarified in our response to commentary no. 7 above.

10. **Table 1 & 2, I think that the information in the last column for "use of painkillers" and "NRS back pain" are swapped.**

    Thank you for notifying about this error, which has been corrected in the new *Table 2*.

11. **Table 3, Could the authors state which was the final model for each variable? Also, it would be helpful to see the variables retained in the final model**

    We acknowledge that variable selection is important, and that models for different outcomes often do not use the same predictors.

    In this study, XGBoost was the final model for all outcomes (ODI, NRS leg pain and NRS back pain) for both LDH and LSS. This was already mentioned in subsection *7.2 Model* development, which listed the hyperparameters for the ODI model, and referred to *Supplementary Table S5* for corresponding information about the NRS models. We have further clarified by adding this information in the revised title of *Table 3 Performance of XGBoost models*

12. **Page 18, Sensitivity analysis: How many variables included in the final models (binary vs continuous) overlapped? Please include a table with this result in the supplementary**

    As mentioned in our response to commentary no. 7 above, we did not build binary models. The classification was derived from the continuous models as explained in the response to commentary no. 7 and thus used the same variables, as explained in commentary no 11 above.

References

1. Prang KH, Karanatsios B, Verbunt E, Wong HL, Yeung J, Kelaher M, et al. Clinical registries data quality attributes to support registry-based randomised controlled trials: A scoping review. Contemp Clin Trials. 2022;119:106843.
2. Clement RC, Welander A, Stowell C, Cha TD, Chen JL, Davies M, et al. A proposed set of metrics for standardized outcome reporting in the management of low back pain. Acta Orthop. 2015;86(5):523–33.
3. Meisenberg G, Williams A. Are acquiescent and extreme response styles related to low intelligence and education? Personality and Individual Differences. 2008;44(7):1539–50.
4. Kaur S, Alhaug OK, Dolatowski FC, Solberg TK, Lønne G. Characteristics and outcomes of patients who did not respond to a national spine surgery registry. BMC Musculoskelet Disord. 2023;24(1):164.
5. Berg B, Gorosito MA, Fjeld O, Haugerud H, Storheim K, Solberg TK, et al. Machine Learning Models for Predicting Disability and Pain Following Lumbar Disc Herniation Surgery. JAMA Netw Open. 2024;7(2):e2355024.

VERSION 2 - REVIEW

**Reviewer**	**2**
**Name**	**Canizares, Mayilee**
**Affiliation**	**UHN, Arthritis Program**
**Date**	**13-Apr-2026**
**COI**	

The authors have addressed my concerns satisfactorily. I have no further questions.
